# Senataxin Plays an Essential Role with DNA Damage Response Proteins in Meiotic Recombination and Gene Silencing

**DOI:** 10.1371/journal.pgen.1003435

**Published:** 2013-04-11

**Authors:** Olivier J. Becherel, Abrey J. Yeo, Alissa Stellati, Evelyn Y. H. Heng, John Luff, Amila M. Suraweera, Rick Woods, Jean Fleming, Dianne Carrie, Kristine McKinney, Xiaoling Xu, Chuxia Deng, Martin F. Lavin

**Affiliations:** 1 Radiation Biology and Oncology Laboratory, Queensland Institute of Medical Research, Brisbane, Australia; 2 School of Chemistry and Molecular Biosciences, University of Queensland, St. Lucia, Australia; 3 School of Medicine, University of Queensland, Brisbane, Australia; 4 University of Otago, Dunedin, New Zealand; 5 QCF Transgenics Laboratory, Queensland Institute of Medical Research, Brisbane, Australia; 6 Dana Farber Cancer Institute, Harvard University, Boston, Massachusetts, United States of America; 7 Mammalian Genetics Section, National Institute of Diabetes and Digestive and Kidney Diseases, National Institutes of Health, Bethesda, Maryland, United States of America; 8 University of Queensland Centre for Clinical Research, University of Queensland, Brisbane, Australia; St. Jude Children's Research Hospital, United States of America

## Abstract

Senataxin, mutated in the human genetic disorder ataxia with oculomotor apraxia type 2 (AOA2), plays an important role in maintaining genome integrity by coordination of transcription, DNA replication, and the DNA damage response. We demonstrate that senataxin is essential for spermatogenesis and that it functions at two stages in meiosis during crossing-over in homologous recombination and in meiotic sex chromosome inactivation (MSCI). Disruption of the *Setx* gene caused persistence of DNA double-strand breaks, a defect in disassembly of Rad51 filaments, accumulation of DNA:RNA hybrids (R-loops), and ultimately a failure of crossing-over. Senataxin localised to the XY body in a Brca1-dependent manner, and in its absence there was incomplete localisation of DNA damage response proteins to the XY chromosomes and ATR was retained on the axial elements of these chromosomes, failing to diffuse out into chromatin. Furthermore persistence of RNA polymerase II activity, altered ubH2A distribution, and abnormal XY-linked gene expression in *Setx^−/−^* revealed an essential role for senataxin in MSCI. These data support key roles for senataxin in coordinating meiotic crossing-over with transcription and in gene silencing to protect the integrity of the genome.

## Author Summary

Ataxia with oculomotor apraxia type 2 (AOA2) caused by a defect in the gene *Setx* (coding for senataxin) is part of a subgroup of autosomal recessive ataxias characterized by defects in genes responsible for the recognition and/or repair of damage in DNA. Cells from these patients are characterized by oxidative stress and are defective in RNA processing and termination of transcription. Recent data suggest that senataxin is involved in coordinating events between DNA replication forks and ongoing transcription. To further understand the role of senataxin, we disrupted the *Setx* gene in mice and demonstrated its essential role in spermatogenesis during meiotic recombination and in meiotic sex chromosome inactivation (MSCI). In the absence of senataxin, DNA double-strand breaks persist, RNA:DNA hybrids (R-loops) accumulate, and homologous recombination is disrupted. Senataxin localised to the XY chromosomes during pachytene. This was dependent on Brca1, which functions early in MSCI to recruit DNA damage response proteins to the XY body. In the absence of senataxin, there was incomplete accumulation of DNA damage response proteins on the XY chromosomes and no MDC1-dependent diffusion of ATR to the broader XY chromatin. The end result was a defect in MSCI, apoptosis, and a failure to complete meiosis.

## Introduction

Ataxia oculomotor apraxia type 2 (AOA2), a severe form of autosomal recessive cerebellar ataxia (ARCA) is characterised by progressive cerebellar atrophy and peripheral neuropathy, oculomotor apraxia and elevated α-fetoprotein [1], [2]. The gene defective in AOA2, *SETX*, is also associated with amyotrophic lateral sclerosis 4 (ALS4), an autosomal dominant juvenile-onset form of ALS [3]. Senataxin shares extensive homology in its putative helicase domain with the yeast, *Saccharomyces cerevisiae* splicing endonuclease 1 protein (Sen1p) which possesses helicase activity and is involved in RNA processing, transcription and transcription-coupled DNA repair [4]. A role for senataxin in DNA repair is supported by the observation that AOA2 patient cells display sensitivity to DNA damaging agents such as H_2_O_2_, camptothecin and mitomycin C and have elevated levels of oxidative DNA damage [5].

Senataxin also plays a role in transcription regulation by its ability to modulate RNA Polymerase II (Pol II) binding to chromatin and through its interaction with proteins involved in transcription [6]. mRNA splicing efficiency, splice site selection, and transcription termination were all defective in senataxin-deficient cells [6]. A recent study has described an additional role for senataxin in transcription elongation and termination [7]. Cells deficient in senataxin displayed an increase in RNA read-through and Pol II density downstream of the Poly(A) site and also exhibited increased levels of R-loops (RNA:DNA hybrids that form over transcription pause sites) formation [8], [9]. The yeast ortholog of senataxin, Sen1p, has also been shown to protect its heavily transcribed genome from R-loop-mediated DNA damage [10]. More recently, a role for senataxin has been described at the interface between transcription and the DNA damage response [11]. They revealed that senataxin forms nuclear foci in S/G2 phase cells and these foci increased in response to DNA damage and impaired DNA replication. These foci disappeared upon resolution of R-loops or after inhibition of transcription. Evidence has also been provided for the association of the yeast ortholog of senataxin, Sen1, with DNA replication forks across RNA polymerase II transcribed genes [12]. These data demonstrate a co-ordinating role for Sen1 between replication and transcription.

We generated a *Setx* knockout mouse model to investigate further the role of senataxin. Our data revealed that this protein is essential for male meiosis, acting at the interface of transcription and meiotic recombination, and also in the process of meiotic sex chromosome inactivation (MSCI).

## Results

### Disruption of the mouse *Setx* gene

**Figure 1:**
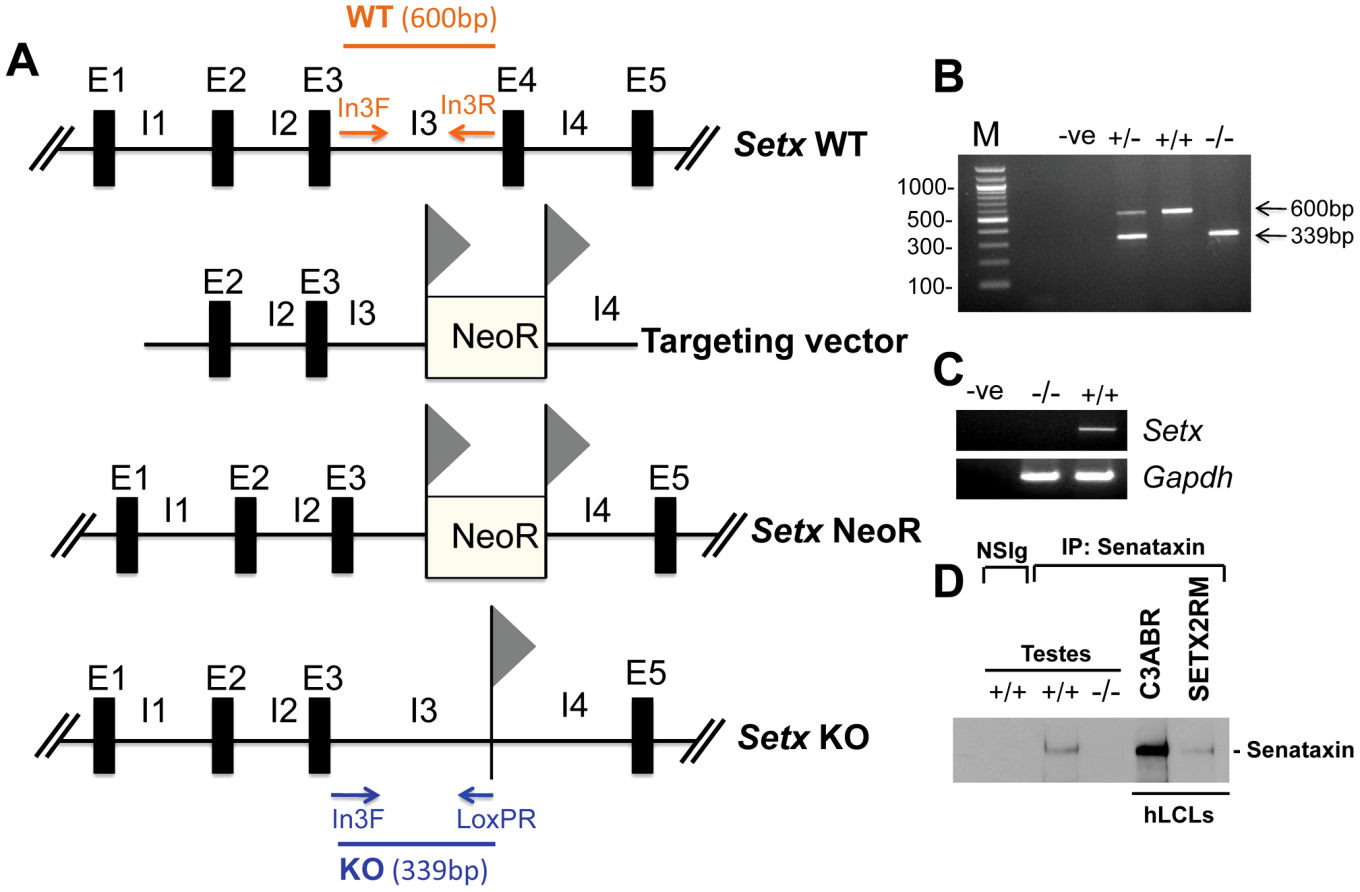
Targeted disruption of the mouse *Setx* gene. A. Diagram of the *Setx* wild type allele (WT), targeting vector, and mutant alleles (neo+ and KO). Primers used for PCR genotyping (In3F, In3R, LoxPR) and the length of the PCR fragments obtained for WT (In3F and In3R yielding a 600 bp product) and KO (In3F and LoxpR yielding a 339 bp product) are indicated. E, exon; I, intron. NeoR represents the neomycin cassette, and triangles the *loxP* sites. B. Representative image of PCR genotyping using In3F, In3R and LoxPR primers. Wild type (+/+), heterozygotes (+/−) and knockout (−/−) alleles generate PCR products of 600 bp, 600 bp and 339 bp, and 339 bp, respectively. A negative control for the PCR reaction (−ve) is also shown. M, 100 bp marker. C. RT-PCR of 35 day-old mice testes samples using primers specific to *Setx* cDNA indicates the absence of *Setx* expression in KO testes. GAPDH was used as an internal standard. D. Immunoprecipitation of senataxin using anti-human senataxin antibodies (Ab-1/Ab-3) from 35 day-old mouse testes extracts confirmed the absence of the protein in the *Setx^−/−^*. Immunoprecipitation of senataxin from human lymphoblastoid cell extracts from normal (C3ABR) and an AOA2 patient (SETX2RM) confirmed the similar size of senataxin in both species. A species-matched non-specific serum (NSIg) was used as a negative control in for the IP experiments. As expected, no senataxin protein was brought down from *Setx* ^+/+^ testes following the IP with the non-specific serum (NSIg).

*Setx^−/−^* mice were produced using a Cre-LoxP system to delete exon 4 as outlined in Figure 1A. Crosses between *Setx* heterozygotes produced all 3 genotypes (Wild type, heterozygotes and homozygote knockouts) as expected (Figure 1B) and a Mendelian inheritance pattern was observed (Wild type 25%; heterozygote 54%; knockout 21%; n = 87). Inactivation of the *Setx* gene was confirmed by RT-PCR and the absence of *Setx* mRNA in the knockout mouse as compared to the wild type (Figure 1C). Immunoprecipitation (IP) with anti-senataxin antibodies from testes extracts confirmed the presence of the protein in *Setx^+/+^* mice but senataxin was not immunoprecipitated from *Setx^−/−^* extracts (Figure 1D). While progressive cerebellar degeneration is characteristic of senataxin-defective AOA2 patients [1], [2] we failed to detect either structural alterations, general cerebellar degeneration, or specific loss of Purkinje cells in *Setx^−/−^* mice (data not shown). Using a simple phenotypic scoring system that has been employed to evaluate mouse models of cerebellar ataxia [13], we failed to reveal any significant neurological/behavioural difference and ataxia between *Setx^+/+^* and *Setx^−/−^* animals (Figure S1).

### Senataxin is essential for germ cell development and fertility

Multiple attempts to breed *Setx^−/−^* mice with each other or with wild type mice were unsuccessful. Male mutant mice had normal development of secondary sexual characteristics, and were capable of the mechanics of mating, but were infertile. Histological examination of *Setx^−/−^* female ovaries at various ages (from 35 days to 8 months of age) revealed no overt phenotypic difference from their wild type littermates, with normal structure and presence of follicles at all stages and an ability to ovulate (Figure S2). However, the yield of viable embryos at 0.5 dpc was very low suggesting that *Setx^−/−^* females are less fertile than their wild type littermates. The fertility of an individual female is a reflection of the number of eggs ovulated and their competence. To investigate the fertility of *Setx^−/−^* female mice, we carried out superovulation and time mating to harvest one-cell stage (0.5 dpc, fertilised egg) embryos in order to compare their viability. A greater than 3.5-fold reduction in the yield of 0.5 dpc for *Setx^−/−^* was observed compared to wildtype animals (10–20 0.5 dpc embryos for *Setx^−/−^* compared to 50–70 for wild types). In addition, only 23% of viable embryos were obtained at 0.5 dpc for *Setx^−/−^* and most of the viable ones did not survive in culture, indicating that *Setx^−/−^* females have a reduced fertility.

**Figure 2:**
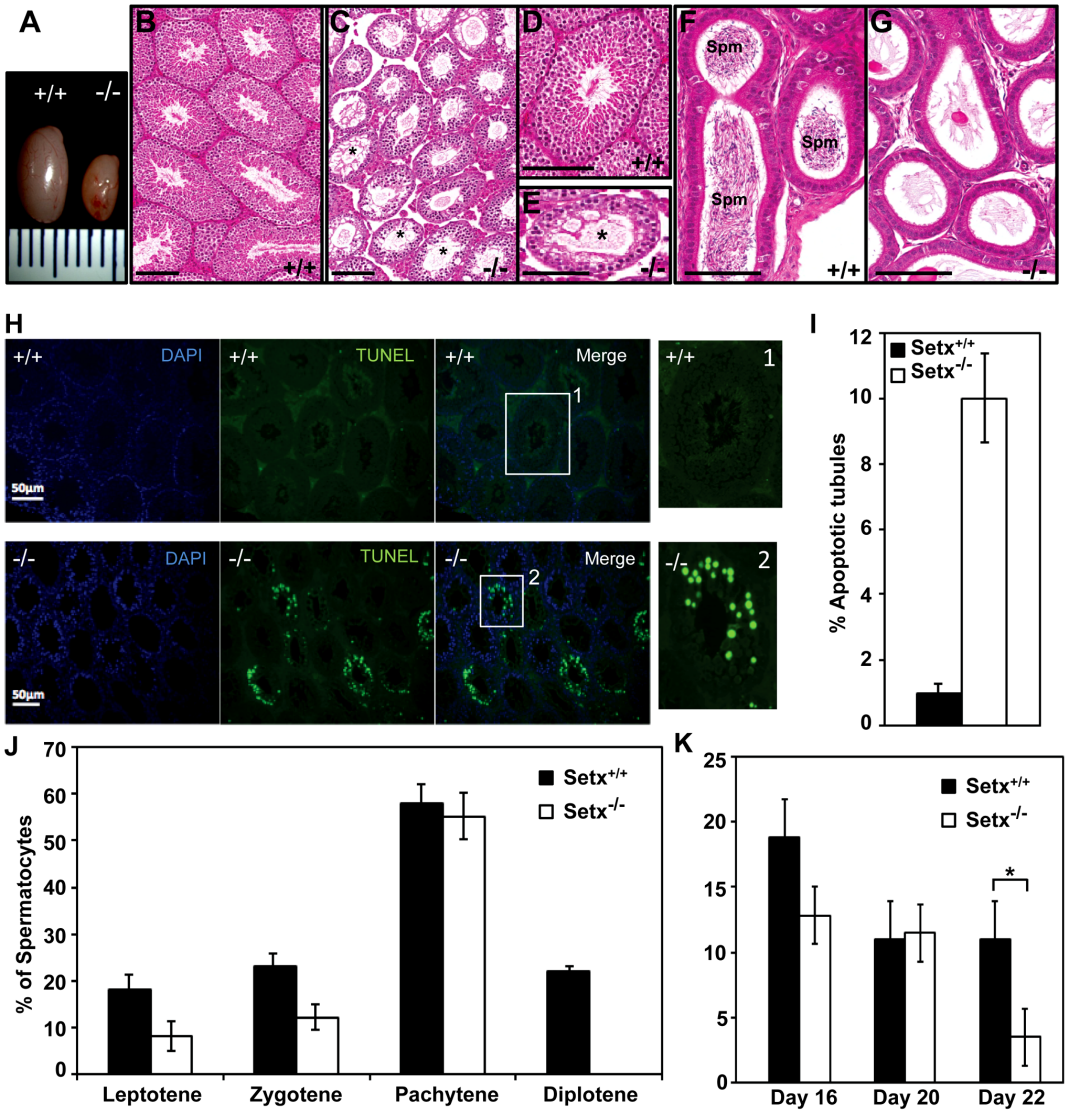
Spermatogenesis is disrupted in *Setx^−/−^* mice. A. Testes from 35-day-old *Setx^+/+^* and *Setx^−/−^* mice. B–C. Hematoxylin and eosin (H&E)-stained sections of testes from adults. Scale bar, 100 µm. D–E. Enlarged images of regions in (B) and (C). Asterisks in (C) and (E) show vacuolated seminiferous tubules in which both spermatozoa and spermatids are absent. Scale bar, 100 µm. F–G. H&E sections of epididymis from *Setx^+/+^* and *Setx^−/−^* adult mice. While there are numerous spermatozoids (Spm) in *Setx^+/+^*epididymis, no sperm is present in *Setx^−/−^*. Scale bar, 100 µm. H. TUNEL-stained sections of testes from adult *Setx^+/+^* and *Setx^−/−^* mice. Many TUNEL-positive cells are observed in *Setx^−/−^* testes. Scale bar, 50 µm. (1) and (2) are magnifications of a seminiferous tubule. I. Increased number of apoptotic tubules in *Setx^−/−^* (Tubules with ≥8 TUNEL-positive cells per tubule). Data is plotted as the mean±standard deviation, n = 1330. J. Block of meiosis at pachytene stage in *Setx^−/−^* mice. Meiotic stage distribution (leptotene, zygotene, pachytene, diplotene) in *Setx^+/+^* and *Setx^−/−^*. While a similar number of leptotene, zygotene and pachytene stage spermatocytes were found in both testes, no diplotene stage spermatocytes were found in *Setx^−/−^* testes indicating a block at pachytene in KO animals. Data plotted as the mean±standard deviation obtained from 3 mice, 2000 spermatocytes were counted in total for both *Setx^+/+^* and *Setx^−/−^*. K. Severe reduction in the number of pachytene spermatocytes in *Setx^−/−^* compared to *Setx^+/+^* during the first meiotic division. No significant change in the percentage of pachytene cells was observed in *Setx^+/+^* from day 16 to 22. However, a significant reduction in the percentage of pachytene spermatocytes was observed at day 22 in *Setx^−/−^* compared to *Setx^+/+^* (Student's t-test, p<0.01). Data plotted as the mean±standard error, n = 6000. * indicates p<0.01.

Since oligospermia and testicular abnormalities are a frequent finding of ARCA patients and the corresponding mouse models [14], [15], we compared the development of testes and seminiferous tubules from *Setx^−/−^* with those from wild type mice. *Setx^−/−^* testes were smaller in size (50–60% reduction in size) than wild-type littermates (Figure 2A) and histological examination of testes from 35 day-old *Setx^−/−^* males revealed a severe disruption of the seminiferous tubules and the absence of germ cells compared to *Setx^+/+^* males (Figure 2B–2G). Morphologically, spermatocytes in *Setx^−/−^* mice appear to have halted development at pachytene stage of meiotic prophase (Figure 2E), suggesting that meiotic arrest in *Setx^−/−^* mice occurs during prophase I. Overall the seminiferous epithelium from an 8-month *Setx^+/−^* mouse testis appears normal but there is evidence of some disruption in places, with few round or elongated spermatids and debris in the lumen (Figure S3). Histological examination of the epididymis from *Setx^−/−^* mice revealed the total absence of mature sperm (Figure 2F–2G) thus confirming the infertility of *Setx^−/−^* males mice. Elevated levels of apoptosis were detected in some tubules of *Setx^−/−^* mice following TUNEL staining (Figure 2H–2I), suggesting that arrested cells are eliminated via this pathway.

To monitor the development of spermatocytes we counted the number of spermatocytes in all stages of meiotic prophase I (Figure 2J). Synaptogenesis appeared to be grossly normal in *Setx^−/−^* mice as determined by staining for synaptonemal complex protein 3 (SCP3) [16] but while the earlier stages of meiosis were represented we failed to detect diplotene stage spermatocytes for *Setx^−/−^*, indicating a block at the pachytene-diplotene transition (Figure 2J). Further analysis of the first meiotic division of prophase I revealed a significant reduction of pachytene spermatocytes from day 16 to day 22 in *Setx^−/−^* (Figure 2K) in line with the lack of diplotene spermatocytes in *Setx^−/−^*. Fragmentation of the synaptomemal complex (SC) at pachytene stage in *Setx^−/−^* was also observed. To investigate the cause of the meiotic defect in more detail, we also determined the expression of spermatogenesis stage-specific markers (Figure S4A) [17], [18]. Expression levels of spermatogonial and early spermatocyte markers *Dmc1*, *Calmegin*, and *A-myb* were similar in both *Setx^−/−^* and *Setx^+/+^*. *Pgk2*, a marker expressed from the beginning (pre-leptotene) and throughout meiosis (leptotene, zygotene, pachytene, diplotene) up to the round spermatid stage showed only a small reduction in expression in *Setx^−/−^* as compared to *Setx^+/+^*. Markers for haploid mature germ cells *Prm1*, *Prm2* and *Tnp1* showed markedly reduced expression in *Setx^−/−^* compared to *Setx^+/+^* (Figure S4B). Together, these data indicate that male germ cells proceed normally from spermatogonia up to the meiotic pachytene stage in *Setx^−/−^* but fail to enter into spermiogenesis and form mature spermatids. Thus, both gene expression of meiosis stage-specific markers and spermatocyte spread analysis confirmed the blockage of meiosis in *Setx^−/−^* male germ cells and indicate that senataxin plays an essential role in the development and maturation of germ cells.

### DNA DSB persist and meiotic crossing-over is defective in *Setx^−/−^*

**Figure 3:**
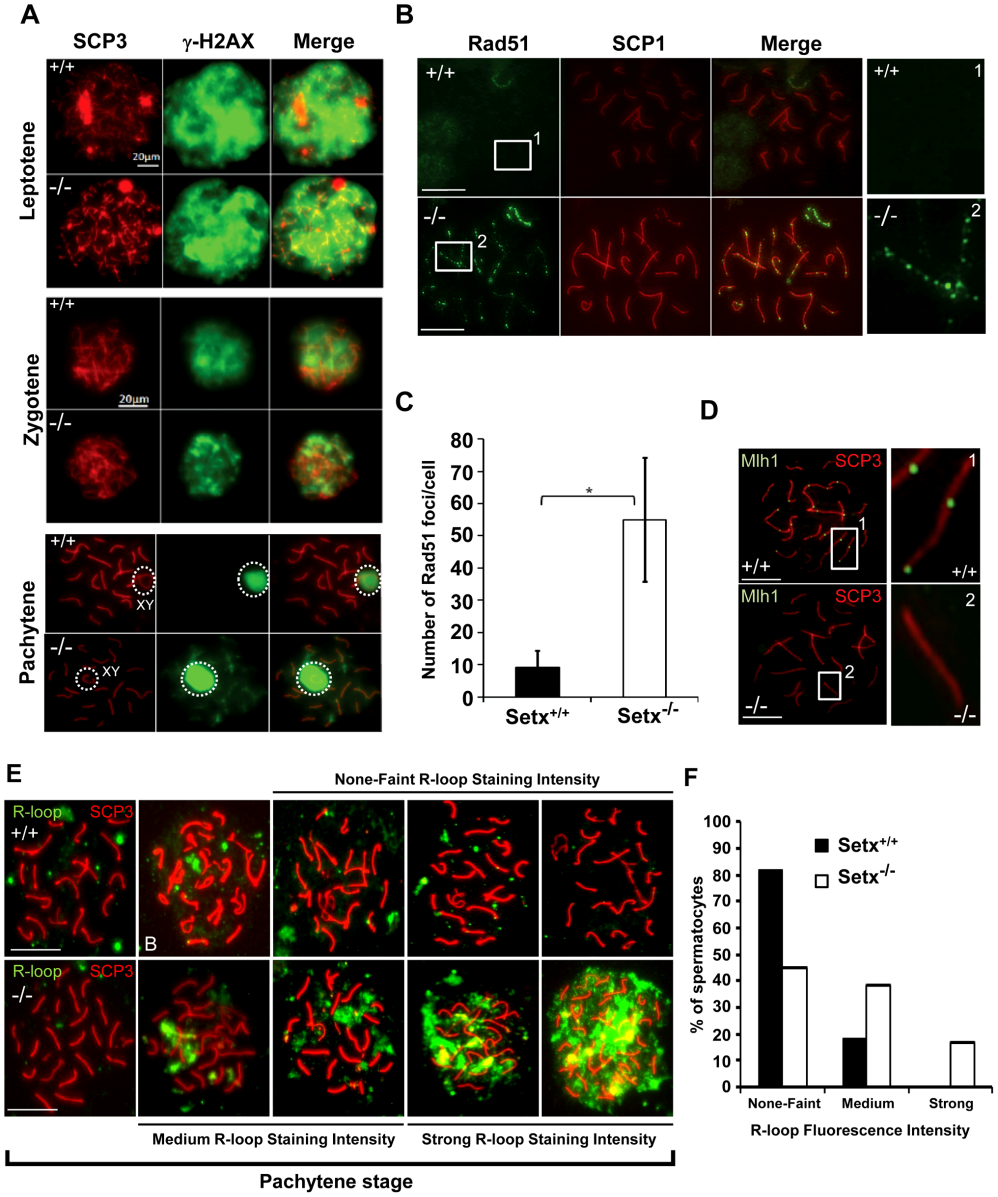
Defective meiotic recombination and crossover formation in infertile *Setx^−/−^* males. A. Initiation and repair of programmed DNA DSB as shown by γH2AX staining of spermatocytes spreads of *Setx^+/+^* and *Setx^−/−^* adult mice. At pachytene, γH2AX staining is restricted to the XY chromosomes (circle) in *Setx^+/+^* spermatocytes, whereas some γH2AX foci remained on asynapsed autosomes indicating persistence of unrepaired DSB in *Setx^−/−^*. Normal γH2AX staining of the XY chromosomes (circle) was observed in both *Setx^+/+^* and *Setx^−/−^* pachytene stage spermatocytes. Scale bar, 20 µm. XY, sex chromosomes. B. Persistence of Rad51 foci at pachytene stage in *Setx^−/−^* spermatocytes indicating the presence of unrepaired DSBs (compare 1 and 2). Scale bar, 20 µm. C. Quantitation of Rad51 foci revealed a 6-fold increase in the number of Rad51 foci at pachytene stage in *Setx^−/−^* as compared to *Setx^+/+^* (Student's t-test, n = 50), * indicates p<0.05. D. Formation of chiasmata at pachytene stage in *Setx^+/+^* spermatocytes as marked by Mlh1 staining. No Mlh1 foci were detected in *Setx^−/−^* pachytene cells indicating that crossovers do not occur in *Setx^−/−^*. 1 and 2 represent magnification of autosomes. Scale bar, 20 µm. SCP3 or SCP1 were used to identify the meiotic stages. E. Defect in senataxin leads to R-loop structures accumulation in germ cells. Staining with S9.6 antibody (R-loops) on adult spermatocytes revealed an increased formation of R-loops in *Setx^−/−^* germ cells. Scale bar, 20 µm. F. Number of pachytene spermatocytes showing none-faint, medium, and strong R-loop staining intensities for *Setx^+/+^* and *Setx^−/−^*.

Meiotic recombination is initiated by the formation of DNA double strand breaks (DSB) catalysed by a type II topoisomerase-like protein Spo11 [19]. These breaks trigger phosphorylation of histone H2AX at ser139 (γH2AX) on large domains of chromatin in the vicinity of the break [20]. As meiosis proceeds to the pachytene stage, γH2AX disappears from synapsed chromosomes and is restricted to the largely unsynapsed sex chromosomes in the sex body [21], [22]. Successful generation of DNA DSB and initiation of repair was observed in *Setx^−/−^* (Figure 3A). At pachytene stage, DSBs disappeared from the autosomes in *Setx^+/+^* mice and only the sex chromosomes stained positive for γH2AX as expected [23], [24]. On the other hand, γH2AX foci remained on apparently synapsed autosomes at pachytene stage in *Setx^−/−^*, indicating the persistence of unrepaired DSBs. Both *Setx^−/−^* and *Setx^+/+^* displayed γH2AX staining at the sex chromosomes at pachytene stage (Figure 3A). Staining of *Setx^−/−^* testes sections for γH2AX confirmed the greater intensity of labelling (Figure S5).

The repair of meiotic DNA DSB occurs via homologous recombination (HR) and involves the participation of various DNA repair factors including RPA, Dmc1 and Rad51 [16]. Both Rad51 and Dmc1 play key roles in the initial steps of HR by mediating strand invasion and homologous pairing. These proteins are normally observed as multiple foci decorating the chromosomes, first appearing at leptotene and sharply decreasing at pachytene [25]. This was the case for Rad51 in *Setx^+/+^* with few foci labelling pachytene chromosomes (Figure 3B). In contrast, multiple Rad51 foci persisted at pachytene stage in *Setx^−/−^* (Figure 3B, Figure S6A), pointing to a defect in Rad51 filament disassembly as a consequence of unrepaired DNA DSB and likely to interfere with HR progression in *Setx^−/−^*. Indeed, quantitation of the number of Rad51 foci at pachytene stage revealed a 6-fold increase of these foci in *Setx^−/−^* compared to *Setx^+/+^* (Figure 3C). This was not due to an over expression of *Rad51* since comparable mRNA levels are observed in both types of mice (Figure S7A). In contrast, immunoblotting of testes protein extracts revealed reduced levels of Rad51 protein in *Setx^−/−^* testes as compared to *Setx^+/+^* indicating that the absence of senataxin is affecting the translation or stability of Rad51 protein (Figure S7B). A similar abnormal pattern of retention at pachytene stage was found for Dmc1 with a 10-fold increase in *Setx^−/−^* compared to *Setx^+/+^* (Figure S6B–S6C). Similar to *Rad51*, comparable levels of *Dmc1* mRNA levels were observed in both mice (Figure S7C). However, we were not able to determine the levels of Dmc1 protein in testes.

To assess whether meiotic recombination is completed in *Setx^−/−^*, we examined the distribution of the mismatch repair protein Mlh1, which normally forms foci and marks the location of chiasmata [26], [27]. We observed an average of 22 Mlh1 foci per pachytene-stage spermatocyte in *Setx^+/+^* (Figure 3D), where up to 78% of spermatocytes SC contain one Mlh1 focus, 19.2% contain 2 foci, 0.5% contain 3 foci and 2.5% had no foci at all, in agreement with previous report [28]. In contrast, no foci were observed in *Setx^−/−^* pachytene-stage spermatocytes (Figure 3D), indicating the absence of crossovers. The lack of Mlh1 foci in *Setx^−/−^* spermatocytes was not due to a defective expression of *Mlh1* gene, as similar levels of the *Mlh1* mRNAs were detected in both *Setx^+/+^* and *Setx^−/−^* testes (Figure S7D). In contrast to Rad51, similar levels of Mlh1 protein in both *Setx^+/+^* and *Setx^−/−^* were shown by Mlh1 immunoblotting of testes protein extracts (Figure S7E). These results confirmed an essential role for senataxin in meiosis.

### Lack of senataxin leads to germ cell accumulation of R-loops and apoptosis

**Figure 4:**
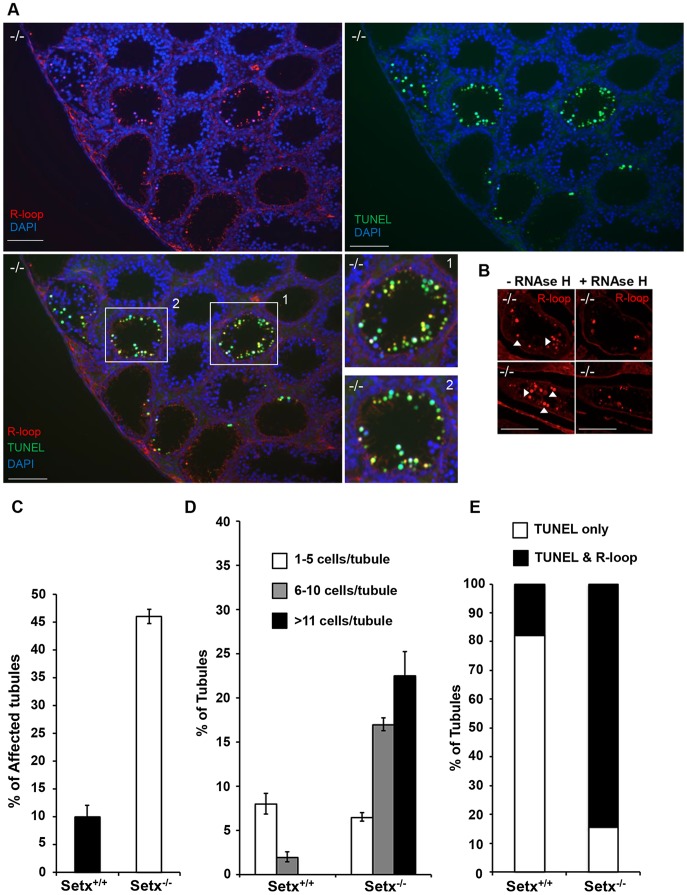
Accumulation of R-loops correlates with apoptosis. A. R-loop and TUNEL co-staining of histological cross-sections of adult *Setx^−/−^* mice revealed apoptosis in germ cells containing R-loops. Hoechst 33342 was used to stain for DNA. Scale bar, 50 µm. B. Pre-treatment of consecutive testes sections from the same animal with RNAse H dramatically reduced R-loop signal intensity in *Setx^−/−^*. Similar results were obtained for consecutive sections from different *Setx^−/−^* animals (data not shown). C. Quantitation of R-loop positive tubules in *Setx^+/+^* and *Setx^−/−^*. The Y-axis represents the % of tubules containing at least one R-loop positive cell within the tubule. D. Higher number of R-loops positive germ cells per tubule (1–5 R-loop-positive cells/tubule, 6–10 R-loop-positive cells/tubule, and more than 11 R-loop-positive cells/tubules) in *Setx^−/−^* compared *Setx^+/+^* confirming the role of senataxin in resolving R-loops *in vivo*. E. Correlation of apoptosis (TUNEL) with R-loop accumulation in *Setx^−/−^* seminiferous tubules uncovers an essential role for senataxin to resolve R-loops and prevent germ cell apoptosis. Graphic representation of the number of tubules that contain TUNEL-only positive germ cells (white) and TUNEL and R-loop co-stained germ cells (black).

Sen1p, the yeast homolog of senataxin was recently found to restrict the occurrence of RNA:DNA hybrids, also known as R-loop structures, that form naturally during transcription, and can trigger genomic instability if left unresolved [10]. Furthermore the same group showed that senataxin resolves R-loop structures to facilitate transcriptional termination in mammalian cells [7]. We reasoned that the defective meiosis in *Setx^−/−^* testes and the consequent apoptosis at pachytene stage might be due to R-loop accumulation as a consequence of transcriptional abnormalities in the absence of senataxin. As shown in Figure 3E, a collection of pachytene-stage spermatocytes from *Setx^−/−^* mice showed a marked accumulation of R-loops compared to *Setx^+/+^*. There was some variation in the R-loop-specific (S9.6) antibody [29], [30] staining intensity between individual pachytene-stage spermatocytes of *Setx^−/−^*, indicating heterogeneity in R-loop accumulation (Figure 3E–3F). The fluorescence intensity of individual pachytene-stage spermatocytes detected by the R-loop-specific antibody was classified into three categories: faint-none, medium and strong as indicated in Figure 3E. No pachytene spermatocytes with strong R-loop staining intensity were observed in *Setx^+/+^* (Figure 3E). This was confirmed with *Setx^−/−^* testes sections which again showed very intense R-loop staining which was variable in different spermatocytes (Figure 4A). Pre-treatment of testes sections with RNAse H prior to immunostaining reduced dramatically the staining intensity in *Setx^−/−^* confirming that these were indeed R-loops (Figure 4B). Co-staining with TUNEL revealed that most cells with accumulated R-loops also undergo apoptosis (Figure 4A, 4E). Although occasionally present in *Setx^+/+^*, R-loop accumulation was dramatically increased in *Setx^−/−^* seminiferous tubules as shown by an increase in the number of R-loop positive cells per tubules (Figure 4C–4D). Co-staining with TUNEL revealed that most cells undergoing apoptosis in *Setx^−/−^* had accumulation of R-loops (Figure 4E). These data suggest that failure to resolve R-loops is responsible for the accumulation of DNA DSB and disruption of meiosis in *Setx^−/−^*.

### Senataxin localises with DNA damage response proteins to the sex chromosomes

**Figure 5:**
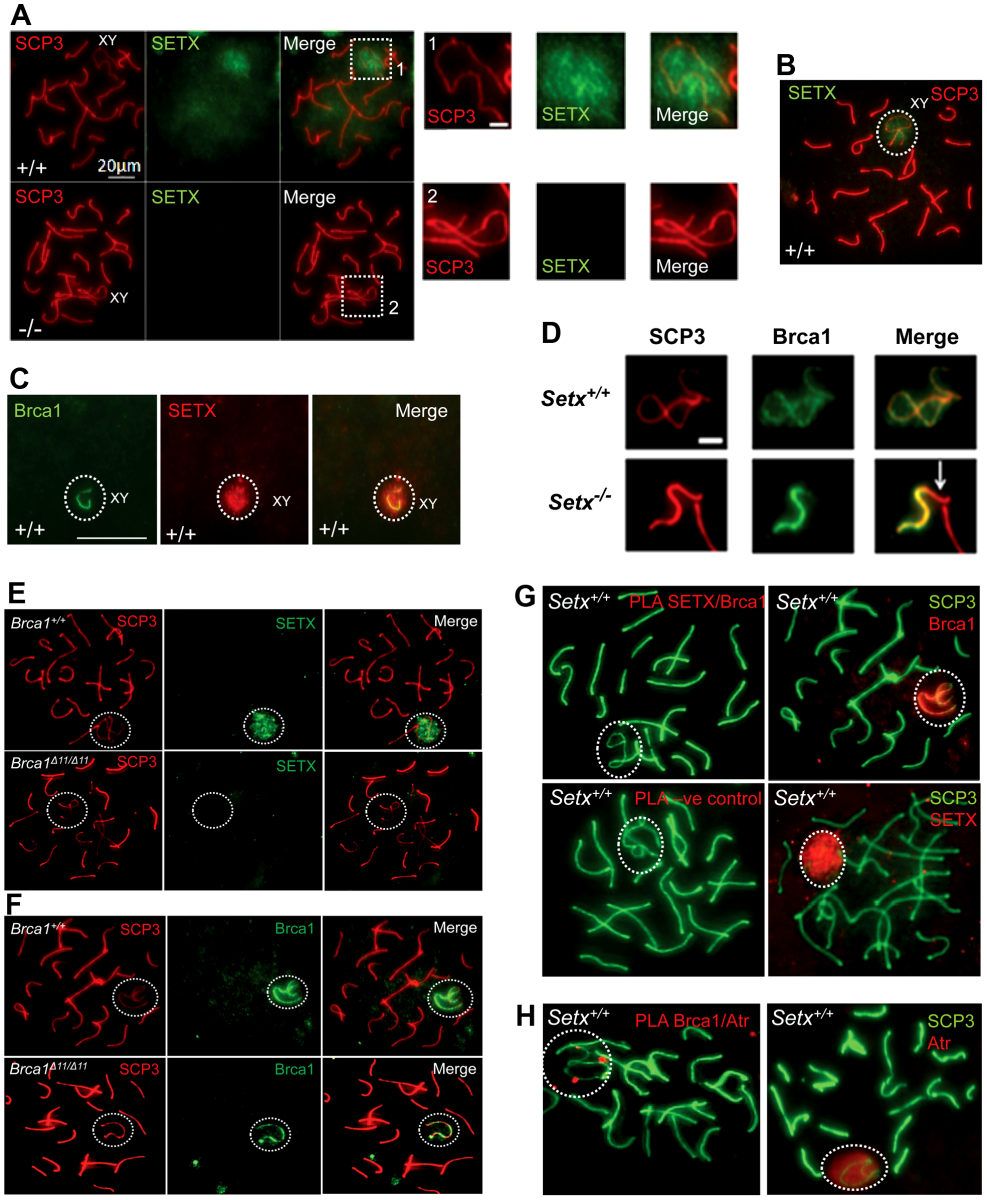
Senataxin localises to the sex chromosomes during meiosis. A. Staining of spermatocytes spreads with an anti-senataxin antibody (Ab-1) revealed that senataxin localised in majority to the XY chromosomes (1) at pachytene stage in *Setx^+/+^*. Some background staining was also observed on the autosomes. No senataxin was detected in *Setx^−/−^* spermatocytes confirming the specificity of our senataxin antibody. (1) and (2) are magnification of the XY chromosomes. B. Double staining of *Setx^+/+^* pachytene spermatocytes with senataxin (Ab-1) and SCP3 revealed a diffuse localisation of senataxin to the XY body. Scale bar, 20 µm. C. Partial co-localisation of senataxin with the XY chromosome marker Brca1. While Brca1 stains exclusively the unsynapsed axis of the XY chromosomes, senataxin staining is more diffuse. D. Brca1 staining of the XY chromosomes in *Setx^+/+^* and *Setx^−/−^* pachytene spermatocytes (day 20). In *Setx^+/+^*, the unsynapsed axis of the XY chromosomes is entirely stained with Brca1 while an incomplete covering (white arrow) of the XY chromosomes is observed in *Setx^−/−^*. Scale bar, 5 µm. E. Lack of senataxin recruitment to XY chromosomes in *Brca1^Δ11/Δ11^ p53^+/−^* as compared to *Brca1^+/+^ p53^+/−^*. F. Brca1 localised to only part of the unsynapsed axis of the XY chromosomes in *Brca1^Δ11/Δ11^ p53^+/−^* while Brca1 coated the entire unsynapsed axis of the XY chromosomes in *Brca1^+/+^ p53^+/−^*. G. Lack of evidence for an *in situ* direct endogenous interaction between senataxin and Brca1 on the XY chromosomes as revealed by negative Proximity Ligation Assay (PLA) results on pachytene spermatocyte spreads. Immunostaining of *Setx^+/+^* pachytene spermatocytes with Brca1 and Setx individually with SCP3 is also shown. H. Endogenous interaction between ATR and Brca1 was confirmed by PLA. Here, we reveal for the first time a direct endogenous interaction between Brca1 and ATR *in situ* over the XY chromosomes.

To investigate in more detail the role of senataxin in meiosis, we studied its localization by performing immunostaining on *Setx^+/+^* spermatocyte spreads. As shown in Figure 5A–5B, senataxin localised mostly to the sex chromosomes at pachytene stage. Some background staining was also observed over the autosomes (Figure 5A) in line with its effect on meiotic recombination and R-loop resolution. As expected there was no senataxin labelling in *Setx^−/−^* spreads (Figure 5A). Partial co-localisation between senataxin and Brca1 was observed albeit there was a more diffuse distribution of senataxin in the XY body (Figure 5C). Brca1 labels the axis of unsynapsed sex chromosomes at pachytene stage to where it is recruited to initiate (MSCI) meiotic sex chromosome inactivation [22]. While Brca1 localised to the axis of the sex chromosomes in *Setx^−/−^* this was incomplete since it was excluded from part of the chromosome (Figure 5D). It appears that this corresponds to the Y chromosome based on the structural morphology [31]. We also determined whether there was a dependence on Brca1 for localisation of senataxin to the sex chromosomes using a *Brca1^Δ11/Δ11^ p53^+/−^* mutant mouse. The results in Figure 5E show that while senataxin localises to XY chromosomes in wild-type mice it fails to do so in Brca1 mutant mice. The Brca1^Δ11/Δ11^ mutant protein still localises to the sex chromosome but is unable to recruit senataxin (Figure 5F). We next determined whether senataxin and Brca1 interacted using Brca1 and senataxin co-immunoprecipitations from testes extracts. We failed to co-immunoprecipitate endogenous senataxin and Brca1 from mouse testes extracts (data not shown). In addition, Proximity Ligation Assay (PLA) which allows for the *in situ* detection of endogenous protein-protein interactions failed to reveal a direct interaction between these two proteins (Figure 5G). In contrast, we confirmed the previously reported endogenous Brca1 and ataxia-telangiectasia and Rad3 related (ATR) interaction [22] using PLA *in situ* over the XY body (Figure 5H). A specific PLA signal for the Brca1/ATR interaction is observed on/around the axis of the unsynapsed XY chromosomes in *Setx^+/+^* pachytene spermatocytes in agreement with the Brca1 and ATR distribution patterns over the sex chromosomes.

**Figure 6:**
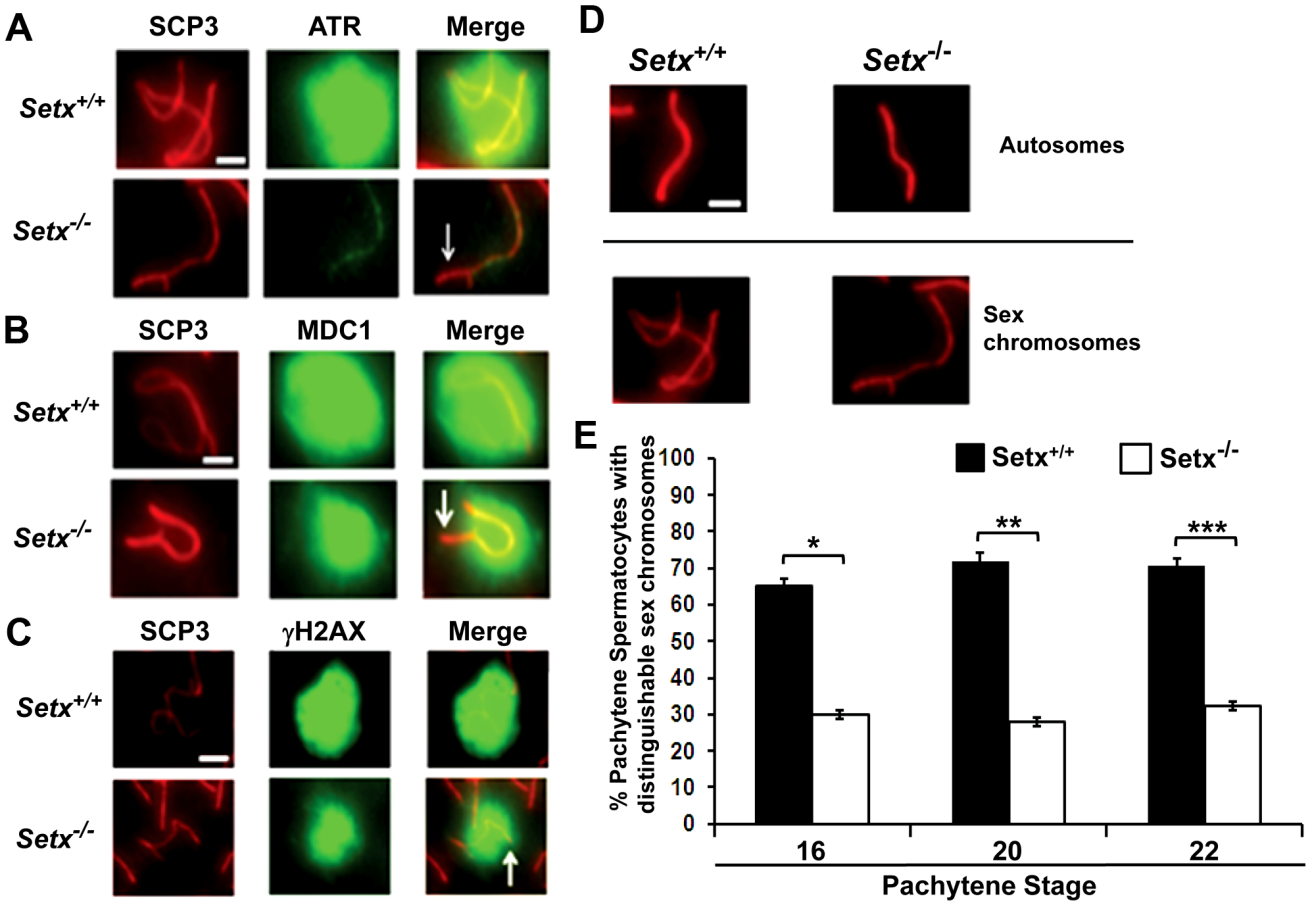
Defective localisation and diffusion of DNA damage response proteins in *Setx^−/−^*. A. Absence of ATR diffusion over the XY chromatin domain in *Setx^−/−^* compared to *Setx^+/+^*. Scale bar, 5 µm. B. Incomplete diffusion of MDC1 over the XY chromatin domain in *Setx^−/−^*, as indicated by the white arrow. Scale bar, 5 µm. C. Reduced intensity and diffusion of γH2AX staining on the XY chromosomes in *Setx^−/−^* compared to *Setx^+/+^*. D. Altered XY chromosomes structure and formation in *Setx^−/−^* as shown by SCP3 staining. Scale bar 5 µm. E. Percentage of *Setx^+/+^* and *Setx^−/−^* pachytene spermatocytes at days 16, 20 and 22 with clearly distinguishable XY chromosomes. At every time point, a significant higher percentage of distinguishable XY chromosomes was observed in *Setx^+/+^* (p<0.01 according to Student's t-test, n = 3600). *,**,*** indicates p<0.01.

At pachytene stage, ATR kinase, another marker of XY chromosomes, is recruited to the unsynapsed axis of the XY chromosomes through an interaction with Brca1 and then diffuses to XY chromatin where it phosphorylates serine 139 of histone H2AX to trigger chromosomal condensation and transcriptional silencing [22], [32]. The results in Figure 6A show a diffuse staining pattern for ATR in *Setx^+/+^* on the XY body. On the other hand, ATR decorates only part of the XY chromosome in *Setx^−/−^* and does not diffuse out into chromatin (Figure 6A and Figure S8). The mediator of DNA damage 1 (MDC1) protein also plays a key role at this stage in MSCI [33]. Recognition of unsynapsed axis of the XY chromosomes by Brca1, ATR and TopBP1 is independent of MDC1 but the chromosome wide spreading of these proteins is dependent on MDC1. We observed that MDC1 labelled the X chromosome but as with Brca1 and ATR failed to decorate the complete XY chromosome (Figure 6B). However, γH2AX labelling was localised to XY chromatin (Figure 6C). As spermatocytes progress from early to mid pachytene the X chromosome appears elongated and sickle shaped prior to loop “curled bundle” formation in late pachytene [31]. These looped XY structures were observed in *Setx^+/+^* but sickle shaped chromosomes appeared mostly in *Setx^−/−^* indicative of arrest in mid pachytene (Figure 6D). Distinguishable “curled bundle” sex chromosomes in *Setx^−/−^* pachytene spermatocytes were seen only in half the percentage of wildtype (Figure 6E). Thus, the absence of senataxin also affects XY body formation/structure and reveals a defect in the recognition and distribution of DNA damage response proteins on the sex chromosomes and thus results in MSCI failure.

### Defective meiotic sex chromosome inactivation (MSCI) in *Setx^−/−^* spermatocytes

**Figure 7:**
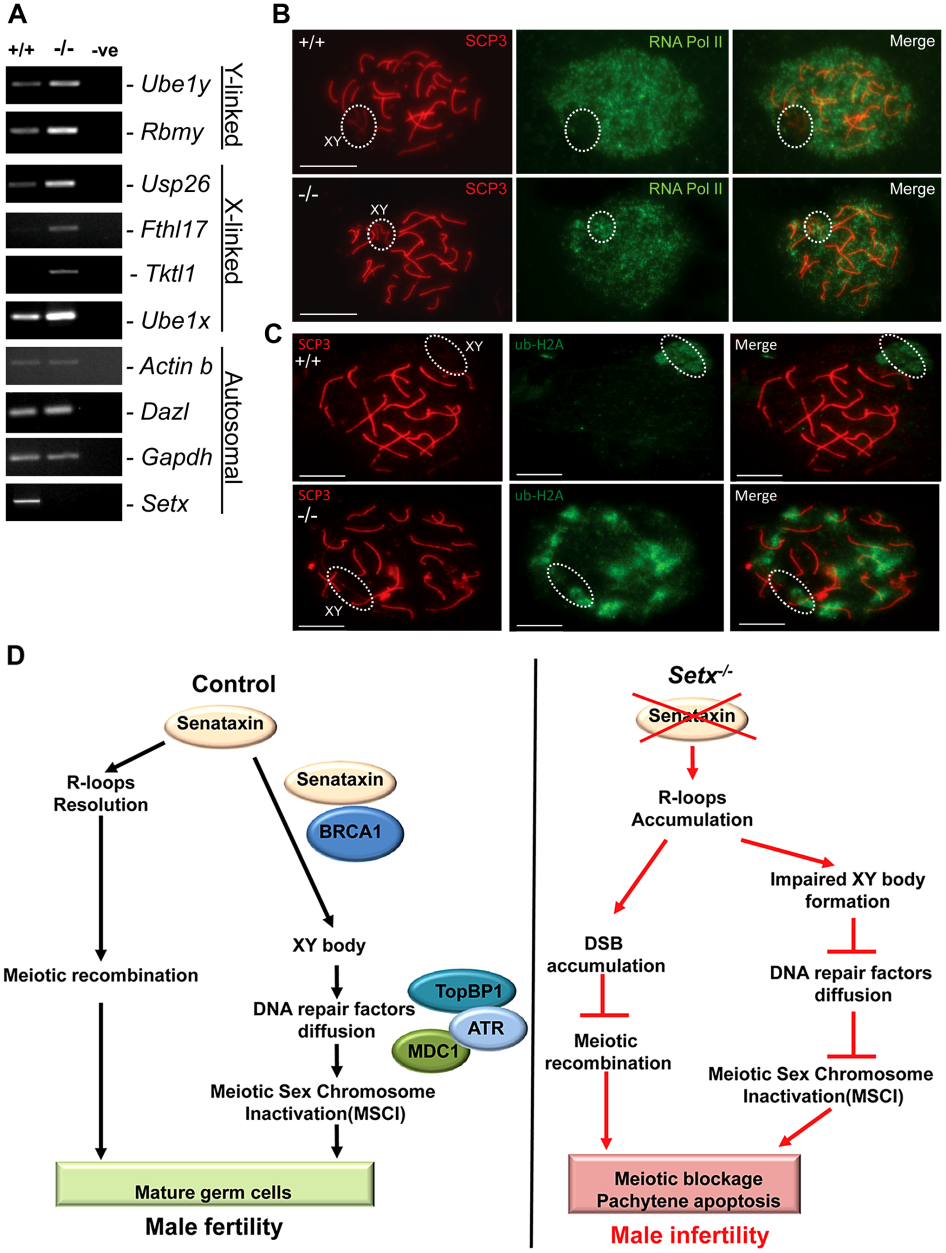
Aberrant meiotic sex chromosome inactivation in *Setx^−/−^* mice. A. Relative levels of transcript of XY-linked germ cell specific genes from *Setx^+/+^* and *Setx^−/−^* testes was determined by RT-PCR to assess MSCI as previously described [35]. Levels of *Actb* and *Gapdh* mRNA were used as positive controls for ubiquitous gene expression. Levels of *Dazl* transcripts were determined as a positive control for previously documented autosomal meiotic expression [35]. Levels of *Ube1x* and *Ube1y* transcripts were included as positive controls for previously documented X- and Y-linked gene expression during spermatogenesis, respectively [35]. X-linked *Usp26*, *Fthl17* and *Tktl1*, and Y-linked *Rbmy* genes have previously shown evidence of MSCI [35]. RT-PCR control without template is also shown. B. Immunostaining for the transcriptionally-active form of RNA Pol II (phospho-S2) revealed active transcription of the XY chromosomes in *Setx^−/−^* spermatocytes (circle). In contrast, no signal for active Pol II was visible over the XY chromosomes in *Setx^+/+^* spermatocytes (circle) confirming the silencing of the XY chromosomes. Scale bar, 20 µm. C. Immunostaining for ubiquitinated histone H2A (ubi-H2A) in *Setx^+/+^* and *Setx^−/−^* spermatocytes reveals a specific staining on the XY chromosomes (circle) in *Setx^+/+^*. In contrast, while ubi-H2A staining was still present on the XY chromosomes in *Setx^−/−^* (circle), it was also distributed on the autosomes. Scale bar, 20 µm. D. Model depicting the consequences of senataxin disruption on spermatogenesis. In wild type spermatocytes, senataxin resolves R-loops structures that may form during pachytene following the resumption of transcriptional activity, thus allowing effective meiotic recombination to proceed and spermatogenesis to be completed. Senataxin also localizes to the XY chromosomes in a Brca1-dependant manner thus enabling the localization of DNA damage response proteins MDC1, ATR and TopBP1 to the entire XY chromatin domain. This ensures that MSCI takes place and germ cells develop and mature properly. On the other hand, in *Setx^−/−^* spermatocytes, R-loops accumulates at pachytene stage interfering with meiotic recombination and the repair of DNA DSB. This leads to the absence of crossing-over, the arrest of meisois at pachytene stage and the elimination of *Setx^−/−^* spermatocytes by apoptosis. The absence of senataxin also results in impaired XY body formation, defective localization of DNA damage response proteins on XY chromatin and failure to undergo MSCI. The combined defects of aborted meiotic recombination and MSCI failure are responsible for the infertility observed in *Setx^−/−^* males.

In mammalian spermatogenesis, the sex chromosomes are transcriptionally-silenced during the pachytene stage of meiotic prophase I, forming a condensed chromatin domain termed the sex body [31], [34]. In the majority of Brca1 mutant pachytene cells sex bodies do not form and transcription is maintained, demonstrating a failure in MSCI [22]. To determine whether the absence of senataxin had a similar effect on MSCI, we analysed the expression of sex-linked genes in *Setx^−/−^* mice using RT-PCR as previously described [35], [36]. As shown in Figure 7A, an increase in the expression of the X-linked *Usp26* (2.44 fold), *Fthl17* (1.4 fold), *Tktl1* (1.36 fold) and *Ube1x* (1.65 fold) genes was observed for *Setx^−/−^* mice compared to *Setx^+/+^* mice. This was also true for several Y-linked genes that include *Ube1y* (2 fold) and *Rbmy* (2.14 fold) indicating that MSCI is defective in *Setx^−/−^*. Normal expression for autosomal genes *Actinb*, *Dazl*, and *Gapdh* was also observed, confirming the specific nature of MSCI (Figure 7A). In order to confirm that MSCI was induced, staining for the activated form (Phospho-S2) of RNA polymerase II (Pol II), which is engaged in transcriptional elongation, revealed a lack of staining at the XY body in *Setx^+/+^*, confirming transcriptional silencing (Figure 7B). In contrast, Pol II staining was visible over XY chromosomes in *Setx^−/−^* (Figure 7B). Ubiquitination of histone H2A has been shown to be associated with transcriptional silencing of large unravelled chromatin regions of the XY chromosomes [37]. Because of the continued presence of RNA Pol II on the sex chromosomes in *Setx^−/−^* we predicted that ubiquitination of H2A would be defective in *Setx^−/−^*. The results in Figure 7C revealed marked localisation of ubi-H2A to the XY body in *Setx^+/+^* spermatocytes. On the other hand the extent of ubi-H2A on the XY body of *Setx^−/−^* was much reduced but ubi-H2A was also distributed across the autosomes. These data suggest that senataxin plays a key role in the initial Brca1-dependent stage in MSCI.

## Discussion

This study provides compelling evidence for an essential role for senataxin in spermatogenesis. We showed that senataxin removes R-loops to maintain the integrity of the genome during meiotic recombination and it is also required for effective MSCI. In *Setx^−/−^* mutant mice, spermatogenesis was arrested in pachytene stage where R-loop accumulation in cells coincided with apoptosis, resulting in male infertility. Testicular atrophy, depletion of germ cells and sterility are common features of animal models with defects in meiotic proteins such as Spo11 [38], strand exchange protein Dmc1 [39], Brca1 [40] and mismatch repair proteins Msh4, Msh5, Mlh3 and Mlh1 [41]–[43]. The phenotype in *Setx^−/−^* male mice overlaps with but is distinct from that described for these mutant mice. Unlike that for *Setx^+/+^*, where breaks were confined to the XY body in pachytene, breaks were still present in the autosomes as well as the XY body in *Setx^−/−^* mice indicating a defect in repair of DNA DSB and consequently a defect in meiotic recombination. This was confirmed by persistence of Rad51 and Dmc1 on autosomes and a failure to detect chiasmata at late meiotic nodules in *Setx^−/−^* pachytene cells. Failure to remove Rad51, as seen in *Setx^−/−^*, prevents the completion of meiotic DSB repair. The meiotic phenotype of *Setx^−/−^* mice resembles that seen in *Brca1^Δ11/Δ11^p53^+/−^* mice [44]. In that model, chromosome synapsis occurred normally and cells progressed through to pachytene, however no chiasmata were observed [44]. Furthermore, DSBs were not repaired in the correct temporal framework, as demonstrated by persistent γH2AX foci. While the failure to complete meiosis due to persistence of unrepaired DSB and lack of cross-overs is common to the *Setx^−/−^* and *Brca1* mutants, one obvious difference is diminished numbers of Rad51 foci and normal localisation of Dmc1 in the *Brca1* mutant [44] This could be accounted for by the interaction of Rad51 with Brca1 which would be disrupted in the *Brca1* mutant.

Sen1, the yeast homolog of senataxin, restricts co-transcriptionally formed R-loops which accumulate in *sen1-1* mutant in a transcription-dependent manner [10]. Furthermore, Mischo *et al* [10] observed a genetic interaction between *sen1* and various factors involved in HR such as *rad50*, *mre11*, *sgs1* and *rad52* and concluded that sen1 plays a pivotal role in preventing genomic instability by transcription-mediated recombination [10]. More recently, Skourti-Stathaki *et al* [7] provided evidence that senataxin, like sen1, resolves R-loop structures formed at transcriptional pause sites to ensure effective transcription termination. *In vivo* accumulation of R-loops was evident in *Setx^−/−^* seminiferous tubules and in pachytene stage spermatocytes, supporting a role for senataxin in resolving such structures. Furthermore, partial co-localisation between R-loops and TUNEL staining in *Setx^−/−^* germ cells indicates that this accumulation may contribute to cell death (Figure 4A–4E). Transcriptional R-loop formation in eukaryotes is highly correlated with DNA recombination and/or impairment of genome stability, indicating an inherent impact of R-looping on the integrity of the genome [9], [45]. R-loop formation is capable of inducing hyper-recombination and/or hypermutation phenotypes in eukaryotes [8], [4]. Recently, THO mutants from *S. cerevisiae* and *C. elegans* showed defective meiosis and an impairment of premeiotic replication as well as DNA-damage accumulation [46]. Gan et al. [47] have shown that R-loop formation impairs DNA replication which is responsible for the deleterious effects of those structures on genome stability. More recently, Alzu et al. [12] provided evidence that when transcription and replication collide Sen1 displaces R-loops to counter recombinogenic events. Thus, R-loop formation may be an intrinsic threat to genome integrity throughout evolution and species have evolved a variety of co-transcriptional processes to prevent the formation of these structures [46]. Senataxin represents a novel factor that minimizes the impact of R-loops that arise as part of normal transcription processes [7], [11] and/or DNA-damage-induced transcription stalling [48]. In the case of *Setx^−/−^* spermatocytes, accumulation of R-loops occurs throughout leptotene and zygotene at a time when DNA DSB are being repaired by crossing over and other mechanisms. Consequently it is likely that R-loops collide with Holiday junctions and interfere with resolution of DNA DSB and thus meiotic recombination. Furthermore, the accumulation of R-loops throughout the chromatin would also affect the repair of DNA DSB that are repaired through non crossover mechanisms.

Senataxin specifically localises to the XY body in pachytene stage partially co-localising with Brca1, MDC1 and ATR suggesting that it might have a role in MSCI. While Brca1 lines the unsynapsed axes of the XY chromosomes, senataxin is associated with these chromosomes but also has a more diffused distribution on chromatin. Prior to MSCI initiation, Brca1 is targeted to the unsynapsed axial elements of the X and Y chromosomes where it remains [22]. It subsequently recruits ATR to the axial elements where it phosphorylates H2AX. In agreement with these findings we provided additional evidence for a direct endogenous interaction between Brca1 and ATR *in situ* over the XY body (Figure 5H). It seems likely that recruitment of senataxin is Brca1-dependent since senataxin did not localize to the XY chromosome in *Brca1^Δ11/Δ11^ p53^+/−^* mutant mice even though the smaller protein mutant Brca1 (lacking exon 11) lined the axes of these chromosomes. We did not detect a direct endogenous interaction between senataxin and Brca1, suggesting that the Brca1-dependent localisation of senataxin to the XY chromosome may be indirect and mediated by other DNA damage response proteins involved in MSCI. On the other hand, Brca1 still localised to the sex chromosomes in *Setx^−/−^* mutant mice. However, this was incomplete since it was excluded from part of the XY structure in pachytene. This is consistent with a recent report that the X and Y chromosomes have different patterns of incorporation and release of recombination/repair and MSCI-related factors during different stages of meiosis [31]. In that study, they provided evidence that some MSCI steps are triggered much later on the Y chromosome than the X chromosome. Comparison with these results suggests that Brca1 has not localised to the Y chromosome in *Setx^−/−^* spermatocytes due to a block earlier in pachytene.

Once Brca1 localises to the axial elements of the sex chromosomes it recruits ATR which phosphorylates H2AX and it subsequently diffuses out into XY chromatin to trigger MSCI [22]. In Brca1 mutant cells, these proteins do not localise to the surrounding chromatin [44]. Loss of senataxin did not change the overall distribution of Brca1 on the XY chromosomes but ATR is no longer diffusely distributed and is instead retained on the axial elements of the XY chromosomes, similar to Brca1. The pattern of ATR staining in *Setx^−/−^* suggests that meiosis only proceeds from early to mid pachytene in these mice and that ATR re-localisation is dependent on senataxin (Figure 7D). Recent data show that in the absence of MDC1, the diffusion of ATR, γH2AX and TopBP1 into XY chromatin is defective [33]. In the absence of senataxin the failure of ATR to diffuse from the axial elements to XY chromatin might be explained by defective MDC1 function. Our observation that MDC1 fails to localize fully to the XY body is consistent with this.

During leptotene and zygotene, the sex chromosomes are transcriptionally active [49]. However, at pachytene stage when meiotic synapsis is complete, the sex chromosomes are rapidly silenced and compartmentalized into a peripheral nuclear subdomain, the sex body [50]. MSCI then persists throughout the rest of pachytene and diplotene [49]. The second wave of phosphorylation only occurs on the chromatin of the sex chromosomes and is absolutely essential for MSCI [24], [50]. This second wave of H2AX phosphorylation occurs in *Setx^−/−^* but breaks were still evident in the autosomes. This, together with failure to form chiasmata points to regions of asynapsis in *Setx^−/−^* autosomes. Extensive asynapsis has been shown to result in MSCI failure and pachytene stage IV apoptosis [51], [52]. Expression analysis of X- and Y-linked genes revealed defective MSCI in *Setx^−/−^*. Furthermore, in contrast to that for *Setx^+/+^* mice RNA Pol II staining was still visible on sex chromosomes in *Setx^−/−^*, consistent with continuing transcription. A reduction in ubi-H2A on the XY body of *Setx^−/−^* is also consistent with a failure of MSCI. While no ubi-H2A was observed associated with autosomes in *Setx^+/+^*, in keeping with transcriptional reactivation at pachytene stage, significant staining is seen on *Setx^−/−^* autosomes pointing to widespread abnormalities in transcriptional activity in these cells. This is in agreement with the increased R-loop staining observed at pachytene stage in *Setx^−/−^* cells. Recent results show that Brca1 preferentially mono-ubiquitinates H2A at satellite DNA regions and Brca1 deficiency impairs the integrity of constitutive heterochromatin causing disruption of gene silencing very likely through loss of ubi-H2A [53].

The evidence presented here suggests that R-loops accumulate in *Setx* deficient, actively transcribing cells in the presence of unrepaired DNA DSB. This supports a role for senataxin in resolving R-loops (Figure 7D). However, we previously showed that senataxin has a broader role in RNA processing since splicing efficiency, alternate splicing and transcription termination are abnormal in AOA2 cells [6]. It is unlikely that the extent of R-loops accumulation in actively transcribing/replicating spermatocytes will be duplicated in post-mitotic cells, such as Purkinje Cells. Neither DNA replication nor recombination is taking place in neuronal cells thus avoiding collisions with the transcriptional apparatus. Indeed, we were not able to detect R-loops in the cerebellum and brain of *Setx^−/−^* mice (unpublished data). These data suggest that the major clinical neurodegenerative phenotype seen in AOA2 patients is more likely to be due to a more general defect in RNA processing leading to reduced transcription fidelity rather than a failure to resolve R-loops. Altogether, these findings reveal a complex and coordinated network between transcription, RNA processing, and DNA repair pathways (Figure 7D), and support the emerging importance of RNA processing factors such as senataxin in the DNA damage response.

## Materials and Methods

### Ethics statement

All animal work and experiments have been approved by The Queensland Institute of Medical Research Animal Ethics Committee

### Targeted inactivation of mouse *Setx* gene

To disrupt the *Setx* gene a highly-effective recombineering approach was employed [54]. Briefly, two cassettes, a loxP-F3-PGK-EM7-Neo-F3 (Neo) cassette was inserted into a BAC clone (RP23-389D11), Children's Hospital Oakland Research Institute corresponding to mouse chromosome 2 and covering the *Setx* genomic sequence. The Neo cassette which provides positive selection in ES cells was flanked by a 5′ homology arm of 6.8 kb and a 3′homology arm of 3 kb. ES cells were then transfected with the linearized targeting vector and selected with 150 µg/ml of G418. Successful recombinant ES clones were determined by Southern blotting with a specific probe and PCR genotyping, and targeted cells (+neo) were subsequently micro-injected into C57BL6/129Sv mice blastocysts to generate chimeras. Excision of the Neo cassette was obtained by crossing the chimeras with a *Cre* deleter stain to generate *Setx^−/−^* mice containing only a *LoxP* site.

### Animal husbandry and genotyping

The mice were weaned at 21 days post-partum and ear clipped for identification. Genotyping was carried out by PCR on genomic DNA isolated from tail tips. Tail tips were lysed in directPCR Lysis eagent (Qiagen, USA) as recommended by the manufacturer. The primers used were In3F: 5′-TTTAAGGAACAGTGCTGC-3′, In3R: 5′-ATGAAGCAGGTAGGATT-3′ and LoxPR: 5′-CGAAGTTATATTAAGGGT-3′. PCR Cycling conditions were as follows: 35 cycles, denaturation at 95°C for 30 sec, annealing at 49°C for 30 sec, extension at 72°C for 1 min, with a final cycle and extension of 7 min at 72°C. Two PCR products were generated, a wild-type PCR product of 600 bp, and the targeted PCR product of 339 bp. PCR products were electrophoresed at 100 V for 30 min on 2% TAE Agarose (Boehringer Mannheim, Amresco, Lewes, UK) stained with Ethidium bromide and visualised with UV transillumination using a GelDoc XR (Biorad Laboratories Inc, UK).

### Histological analysis of *Setx* mice testes and ovaries

Testes from adult (35-day-old), 4 months, 8 months and 12 month-old mice were collected and fixed in PBS buffered 10% formalin, embedded in paraffin block and sectioned at 4 µm. Sections were stained with Hematoxylin and Eosin (H&E) and Toluidine blue. Slides were examined under light microscope and then scanned using Scanscope CS system (Aperio Technologies, Vista, USA). Images corresponding to ×10 and ×20 magnification were captured and assembled into Adobe Photoshop 7 (Adobe Systems Inc, USA).

### RT–PCR reactions and gene expression analysis

Total RNA was isolated from 35-day-old wild type and knockout mice testes using the RNeasy mini kit (Qiagen, USA) according to the manufacturer's protocol. RNA concentrations were determined by UV spectrophotometry using a Nanodrop ND-1000 (Thermo scientific, USA). cDNA was made from 5 µg of purified RNA. Briefly, RNA was mixed with 1 µl of random hexamer primers (Bio-Rad Laboratories Inc. USA), 1 µl of 10 mM dNTP mix and DEPC-treated water up to a 14 µl volume. The mixture was heat-denatured at 65°C for 5 min. 4 µl of First Strand buffer (Invitrogen, USA), 1 µl of 1 mM DTT, 1 µl of RNAaseIN (Promega, USA), and 1 µl of SuperScriptIII reverse transcriptase enzyme (Invitrogen, USA) was added to the mixture, and incubated for 10 min at 25°C, then 60 min at 50°C, 15 min at 70°C, and chilled on ice. 1 µl of RNAse H was subsequently added to each tube and incubated for 20 min at 37°C, followed by heat inactivation for 20 min at 65°C. The resulting cDNA were stored at −20°C prior to use. Gene expression analysis was performed by PCR in a 2720 Thermal Cycler (Applied Biosystem, USA). Reactions (25 µl) contained 14.5 µl of sterile water, 50 ng of cDNA template, 1× PCR Buffer II (Roche, Switzerland), 2.5 mM MgCl_2_ (Roche, Switzerland), 20 µM dNTPs, 1 µM of each primer, and 5 µl of AmpliTaq Gold DNA Polymerase (Roche, Switzerland). The primer pairs used for gene expression analysis are described in Table S1. Amplification was for 30 cycles and cycling conditions were as follows: denaturation for 5 min at 95°C for 30 sec, annealing at 55°C for 30 sec, elongation for 1 min at 72°C followed by a final extension step of 7 min at 72°C. PCR reactions were separated on 2% TAE agarose gels and visualised as above.

### Cell extracts and senataxin immunoprecipitation

Testes from 35 day-old mice were collected and ground with a pestle to disrupt their structure and lysed for 1 h at 4°C on a rotating wheel with lysis buffer (50 mM Tris-HCl pH 7.5, 50 mM β-glycerophosphate, 150 mM NaCl, 10% glycerol, 1% Tween 20, 1 mM PMSF, 5 mM DTT and 1× EDTA-free Complete Protease inhibitor (Roche, Switzerland). Cellular debris were pelleted by centrifugation at 16,100×g at 4°C for 10 min, and protein concentration was determined using Lowry Assay (Bio-Rad Laboratories, Inc, USA). 2 mg of total cell extract were pre-cleared with 50 µl of a mixture of 1∶30 protein G+A beads (Millipore, Germany) for 3 hours at 4°C on a rotating wheel. Extract were centrifuged for 5 min at 2000×g, beads were removed, and 20 µg of anti-human senataxin antibody (Ab1/Ab-3) was added to the extract. Extracts and antibody were incubated overnight at 4°C on a rotating wheel to allow binding of the antibody to mouse senataxin. The next day, 50 µl of protein G+A beads were added to the extract and incubated for 1 h at 4°C on a rotating wheel. The immunoprecipitate was subsequently washed 3 times with lysis buffer and the beads were resuspended in gel loading buffer and separated on 5% SDS-PAGE at constant current (20 mA per gel) for 1.5 h. Once separated, proteins were transferred onto a nitrocellulose membrane (Hybond C, Amersham) for 1 h at 4°C with constant voltage (100 Volts). Immunoblotting with anti-senataxin (Ab-1) antibody was performed using standard procedure as previously described [5].

### Spermatocytes spreads, immunostaining, and imaging

All spreads were made from testes collected from adult 35-day-old mice or at day 16, 20 and 22 post partum. Briefly, testes were decapsulated, finely chopped and rinsed in GIBCO DME medium (Invitrogen, USA). Large clumps were removed by centrifugation at 6780×g for 5 min at room temperature. The remaining supernatant was centrifuged to pellet the cell suspension and mixed with 0.1M sucrose and spread onto glass slides pre-wetted with 1% paraformaldehyde and 0.1% Triton X-100 in PBS. Cells were fixed on the glass slides for 2 h at room temperature. The slides were subsequently washed with PBS and air-dried in the presence of a wetting agent, 1∶250 Kodak Photo-Flo 200 (Kodak professional, USA). Once dried, spreads were stored at −80°C. For immunostaining, slides, were rehydrated in dH20, and blocked in blocking buffer (0.2% BSA, 0.2% gelatine in PBS) for 30 min at room temperature. Spreads were incubated with primary antibodies overnight at 4°C in a humidified chamber. Primary antibodies used included anti-SCP3 (1∶100, NB300-230, Novus Biologicals), anti-SCP1-DyeLight conjugated (1∶100, NB300-2201R, Novus Biologicals), anti-γH2AX (1∶100, Y-P1016, Millipore), anti-Rad51 (1∶100, SC-33626, Santa Cruz Biotechnology), anti-Dmc1 (1∶50, 2H12/4, Sapphire Bioscience), anti-Mlh1 (1∶10, G168-15, Sapphire Bioscience), anti-ATR (1∶100, SC-1887, Santa Cruz Biotechnology), anti-senataxin (1∶100, Ab-1, [5]), anti-R-loop (1∶100, S9.6), anti-RNA Pol II (phospho S2) (1∶100, H5, ab24758, Abcam), anti-mouse Brca1 (1∶300, David Livingston), anti-ubi-H2A (1∶100, Clone E6C5, Millipore). Slides were subsequently washes 4 times for 3 min each in PBS on a rocker, and probed with the appropriate Alexa-Dye488 or Alexa-Dye594-conjugated secondary antibodies (1∶250, Invitrogen, Molecular Probes, USA). Slides were washed again 4 times for 3 min each in PBS. DNA was stained with Hoechst 33342 (1∶10,000) for 10 min at room temperature, and slides were mounted in Celvol 603 medium. Images were captured at room temperature using a digital camera (AxioCam Mrm, Carl Zeiss Microimaging Inc., Germany) attached to a fluorescent microscope (Axioskop 2 mot plus, Carl Zeiss Microimaging Inc., Germany) and the AxioVision 4.8 software (Carl Zeiss, Microimaging Inc. Germany). The objective employed was a 63× Zeiss Plan Apochromat 1,4 Oil DIC (Carl Zeiss, Germany). Images were subsequently assembled in Adobe Photoshop 7 (Adobe Systems Inc, USA), and contrast and brightness were adjusted on the whole image panel at the same time.

### TUNEL assay for apoptosis

Terminal deoxynucleotidyl transferase dUTP nick end labeling (TUNEL) is a method for detecting DNA fragmentation by labeling the terminal end of nucleic acids. TUNEL is a common method for detecting DNA fragmentation that results from apoptotic signaling cascades. The assay relies on the presence of nicks in the DNA which can be identified by terminal deoxynucleotidyl transferase (TdT), an enzyme that will catalyze the addition of Fluorescein-labeled dUTP. Paraffin sections were dewaxed and rehydrated with Shandon Varistain Gemini ES (Thermo Scientific, USA). TUNEL assay was performed using the Fluorescence *in situ* Cell Death Detection Kit (Roche, Switzerland) following the manufacturer's instructions. Slides were visualised under a fluorescent microscope and images were captured as previously described. The objective employed was a Zeiss Plan Neofluar ×10/0.30 (×10 magnification). For double staining, TUNEL was carried out first followed by immunostaining as described below.

### R-loop and DNA damage immunostaining on tissue sections

Slides with tissue sections were dewaxed and enzymatic antigen retrieval was performed by incubating the sections with 1∶10 Trypsin dilution in PBS for 20 min at 37°C. Slides were washed 3 times for 5 min with PBS at room temperature for 5 min each. Tissues sections were blocked in (20% FCS, 2% BSA, 0.2% Triton X-100) for 1 h at room temperature. Slides were incubated with anti-R-loop (1∶100, S9.6) [29] or anti-γH2AX (1∶100, Y-P1016, Millipore) antibody overnight at 4°C in a humidified chamber. Slides were washed 5 times with 1× PBS containing 0.5% Triton X-100 for 5 min each at room temperature. Alexa-Dye488 or Alexa-Dye594-conjugated secondary antibody was added for 1 h at 37°C in a humidified chamber. Subsequently, slides were washed 3 times as before and Hoechst 33342 was added for 10 min to staining nuclei. Slides were finally washed twice and glass coverslips were mounted for imaging. Imaging was performed as described above. Confirmation of R-loop specific staining was obtained by pre-treating *Setx^−/−^* testes sections with RNAse H (New England Biolads, USA).

### Proximity Ligation Assay and endogenous *in situ* interaction

To investigate a possible interaction between Brca1, ATR and senataxin we employed *in situ* Proximity Ligation Assay (PLA) (Duolink, Olink Bioscience, Uppsala, Sweden) on wild type (*Setx^+/+^*) spermatocytes spreads. PLA allows the monitoring of protein interactions and modifications with high specificity and sensitivity. Protein targets can be readily detected and localized with single molecule resolution and objectively quantified in unmodified cells and tissues. Utilizing only a few cells, sub-cellular events, such as transient or weak interactions are revealed *in situ*. Two primary antibodies raised in different species recognize the target antigens of interest. Species-specific secondary antibodies, called PLA probes, each with a unique short DNA strand attached to it, bind to the primary antibodies. When the PLA probes are in close proximity, the DNA strands can interact through a subsequent addition of two other circle-forming DNA oligonucleotides. After joining of the two added oligonucleotides by enzymatic ligation, they are amplified via rolling circle amplification using a polymerase. After the amplification reaction, several-hundredfold replication of the DNA circle has occurred, labeled complementary oligonucleotide probes highlight the product. The resulting high concentration of fluorescence in each single-molecule amplification product is visible as a distinct bright spot when viewed with a fluorescence microscope. The assay was performed according to the manufacturer's protocol using rabbit anti-mouse Brca1 (1∶200, David Livingston), sheep anti-senataxin (1∶200, Ab-1) and goat anti-ATR antibody (1∶100, SC-1887, Santa Cruz Biotechnology) antibodies and the corresponding anti-goat PLA Probe MINUS and anti-rabbit PLA probe PLUS. Identification of pachytene stage spermatocytes was determined by counterstaining with SCP3 antibody. PLA was also carried out on *Setx^−/−^* spermatocytes spreads as a negative control. Slide mounting and imaging was performed as described above.

## Supporting Information

Figure S1 Absence of neurological phenotype and ataxia in *Setx^−/−^* mice. Neurological phenotype and ataxia were examined according to the phenotypic scoring system developed by Guyenet et al. 2010 [13]. No gross abnormal behaviour and ataxic phenotype progression was observed over a period of 3 to 17 months. Briefly, this scoring system combines phenotypic assessments that have been previously employed to assess various models of neurological disease including spinocerebellar ataxia, Huntington's disease and spinobulbar muscular atrophy [55]–[57]. Measures include hindlimb clasping, ledge test, gait and kyphosis. Each measure was recorded on a scale of 0–3 (0 representing the absence of the relevant phenotype and 3 the most severe manifestation) with a combined total of 0–12 for all four measures. Ledge test is a direct measure of coordination, the most directly comparable to human signs of cerebellar ataxia, which is impaired in cerebellar ataxias and many other neurodegenerative disorders. Hindlimb clasping is a marker of disease progression in many mouse models of neurodegeneration and certain ataxias [55]. Gait is a measure of coordination and muscle function and kyphosis is a characteristic dorsal curvature of the spine that is commonly observed in mouse models of neurodegenerative diseases [56], [57]. Mice were assessed on a 0–3 scale each for ledge test, clasping, gait and kyphosis. Average composite score for *Setx^+/+^* and *Setx^−/−^* at each age was calculated.
(TIF)

Figure S2 Normal ovary structure in *Setx^−/−^* mice. We examined the histology of *Setx^−/−^* ovaries at 8 months of age to determine whether they exhibited similar signs as in the human patients [1], [2]. A–D: Haematoxylin and eosin-stained 4 µm sections of four 8-month old *Setx^−/−^* mouse ovaries. Pre-antral and antral follicles (F) are present in all sections and many follicles contain oocytes (asterisks). All sections also contain corpora lutea (CL), suggesting ovulation has occurred and the hypothalamic-pituitary-gonadal endocrine axis is intact in these mice. (Arrowheads denote the position of the bursal membrane enclosing the mouse ovary.) Scale bar, 200 µm.
(TIF)

Figure S3 Testis histology of *Setx*-deficient mice. A. Testis of a 35-day old *Setx^+/+^* mouse showing normal testis histology. Scale bar, 200 µm. B. Testis of a 35-day-old *Setx^+/+^* mouse. Scale bar, 100 µm. Interstitial (Leydig) cells lie between the seminiferous tubules (LD). C. Seminiferous tubule from a *Setx^+/+^* mouse at higher magnification. The tubule contains primary spermatocytes undergoing mitosis (short arrow), and elongated spermatids (asterisks) towards the lumen of the tubule, in which few sperm tails (T) can be seen. LD cells lie between the tubules. Sertoli cell nuclei (SCN) are present on the basement membrane of the tubule. D. Testis of a 35-day-old *Setx^−/−^* mouse showing seminiferous tubules with smaller diameter and disrupted spermatogenesis. Scale bar, 200 µm. E. Testis of a 35-day-old *Setx^−/−^* mouse, showing tubules at different stages of the seminiferous epithelial cycle. In some stages, spermatocytes (asterisks) are abundant. In other stages of the seminiferous cycle spermatogonia and some spermatocytes (Sp) line an almost empty tubule. Scale bar, 100 µm F. Seminiferous tubule of a 35-day-old *Setx^−/−^* mouse testis, showing spermatogonia (Spn) and Sertoli cell nuclei (SCN) lining the basement membrane of the tubule. The lumen appears to contain remnants of Sertoli cell cytoplasm. Elongated spermatids and spermatozoa are completely absent. G–J. Male *Setx^+/−^* mice exhibit signs of reduced fertility at 8 months of age. G. Seminiferous tubules contain few mature spermatozoa and large inclusions (arrowheads), suggesting increased apoptosis of spermatogenic cells. The seminiferous epithelium contains post-meiotic round and elongating spermatids. Scale bar, 200 µm. H. Seminiferous tubule from an 8-month *Setx^+/−^* mouse testis; Scale bar, 100 µm. The seminiferous epithelium contains all stages of spermatogenesis, including round (R) and elongating spermatids (asterisks) and there are sperm tails (T) in the lumen. I. Seminiferous tubule from an 8-month *Setx^+/−^* mouse testis. Scale bar, 100 µm. The seminiferous epithelium appears disrupted in places (arrow), with few round or elongated spermatids and debris in the lumen. J. In this seminiferous tubule from 8-month *Setx^+/−^* mouse testis there are few round (R) or elongated spermatids (asterisks) present and the lumen contains cell debris (arrow). Scale bar, 100 µm.
(TIF)

Figure S4 Abortion of meiosis following a block at pachytene stage in *Setx^−/−^* mice. A. Schematic representation of the various stages of spermatogenesis and the temporal expression of stage-specific makers as previously reported [58]. B. Semi-quantitative RT-PCR analysis of spermatogenesis stage-specific markers in *Setx^+/+^* and *Setx^−/−^* testes. Similar levels of expression for mitosis and meiosis-specific markers (*Dmc1*, *Calmegin*, *A-myb*) were observed in both *Setx^+/+^* and *Setx^−/−^* testes. A reduction of *Pgk2* is noticable in *Setx^−/−^*. A marked reduction in expression for post-meiotic germ cells (*Prm1*, *Prm2* and *Tnp1*) is observed in *Setx^−/−^* testes in agreement the absence of these cells in *Setx^−/−^* seminiferous tubules as shown in Figure 2. These data suggest that *Setx^−/−^* spermatocytes do not proceed past meiosis. *Gapdh* was used as an internal standard. Cal, *Calmegin*.
(TIF)

Figure S5 Elevated levels of DSBs in adult *Setx^−/−^* testes sections. γH2AX staining of adult testes histological sections highlight the extensive amount of DNA DSB breaks in *Setx^−/−^* seminiferous tubules.
(TIF)

Figure S6 Persistance of Rad51 and Dmc1 foci at pachytene stage in *Setx^−/−^* and *Brca1^Δ11/Δ11^p53^+/−^ spermatocytes*. A. Persistence of DSB repair intermediates in pachytene cells of *Setx^−/−^* mice. Normal Rad51 foci formation occurred at leptotene and zygotene stage in *Setx^+/+^* and *Setx^−/−^* mice, there was persistence of Rad51 foci at pachytene stage in *Setx^−/−^* spermatocytes indicating the presence of unrepaired DSBs. Scale bar, 20 µm. B. Persistence of DSB repair intermediates in pachytene cells of *Setx^−/−^* mice. Normal Dmc1 foci formation occurred at leptotene and zygotene stage in *Setx^+/+^* and *Setx^−/−^* mice, there was persistence of Dmc1 foci at pachytene stage in *Setx^−/−^* spermatocytes indicating the presence of unrepaired DSBs (compare 1 and 2). Scale bar, 20 µm. C. Quantitation of Dmc1 foci at pachytene stage in *Setx^+/+^* and *Setx^−/−^* mice. A 10-fold increase of Dmc1 foci was observed in *Setx^−/−^*. (Student's t-test, n = 50) * indicates p<0.05. D. Rad51 foci in *Brca1^+/+^p53^+/−^* and *Brca1^Δ11/Δ11^p53^+/−^*. Normal Rad51 foci formation occurred at leptotene and zygotene stage in *Setx^+/+^* and *Setx^−/−^* mice, there was persistence of Rad51 foci at pachytene stage in *Brca1^Δ11/Δ11^p53^+/−^* spermatocytes indicating the presence of unrepaired DSBs. Scale bar, 20 µm. E. Quantitation Rad51 foci at pachytene stage in *Brca1^+/+^p53^+/−^* and *Brca1^Δ11/Δ11^p53^+/−^* spermatocytes. A 2.5-fold increase in the numbers of Rad51 foci was observed in *Brca1^Δ11/Δ11^p53^+/−^* (Student's t-test, n = 50). * indicates p<0.05.
(TIF)

Figure S7 Expression levels of recombination factors. A. RT-PCR analysis of *Rad51* expression revealed that the increased number of Rad51 foci in *Setx^−/−^* is not due to an increased expression of *Rad51* gene since similar levels of *Rad511* mRNA levels were detected in *Setx^+/+^* and *Setx^−/−^* testes. B. Immunoblotting of *Setx^+/+^* and *Setx^−/−^* testes protein extracts with anti-Rad51 antibody shows reduced levels of Rad51 protein in *Setx^−/−^*. Anti-GAPDH was used as a loading control. C. RT-PCR analysis of *Dmc1* expression revealed that the increased number of Dmc1 foci in *Setx^−/−^* is not due to an increased expression of *Dmc1* gene since similar levels of *Dmc1* mRNA levels were detected in *Setx^+/+^* and *Setx^−/−^* testes. D. RT-PCR analysis of *Mlh1* expression revealed that the absence of Mlh1 foci in *Setx^−/−^* is not due to a lack of expression of *Mlh1* gene since similar levels of *Mlh1* mRNA levels were detected in *Setx^+/+^* and *Setx^−/−^* testes. E. Immunoblotting of *Setx^+/+^* and *Setx^−/−^* testes protein extracts with anti-Mlh1 antibody shows similar levels of Mlh1 protein. Anti-GAPDH was used as a loading control.
(TIF)

Figure S8 Abnormal localisation of ATR in *Setx^−/−^* pachytene spermatocytes. ATR foci formation occurred at leptotene and zygotene stages in *Setx^+/+^* and *Setx^−/−^* spermatocytes, however, ATR failed to spread to XY chromatin domain in *Setx^−/−^* at pachytene stage. Scale bar, 20 µm.
(TIF)

Table S1 Sequences of primers used for the gene expression analysis. Sequences of the primers used for the spermatogenesis stage specific markers and X- and Y-linked gene expression analysis.
(PDF)
