# Supplementary figures and images for: Senataxin Plays an Essential Role with DNA Damage Response Proteins in Meiotic Recombination and Gene Silencing
Source: PLoS Genet. 2013 Apr 11;9(4):e1003435. doi: 10.1371/journal.pgen.1003435 (PMC3623790; doi:10.1371/journal.pgen.1003435)

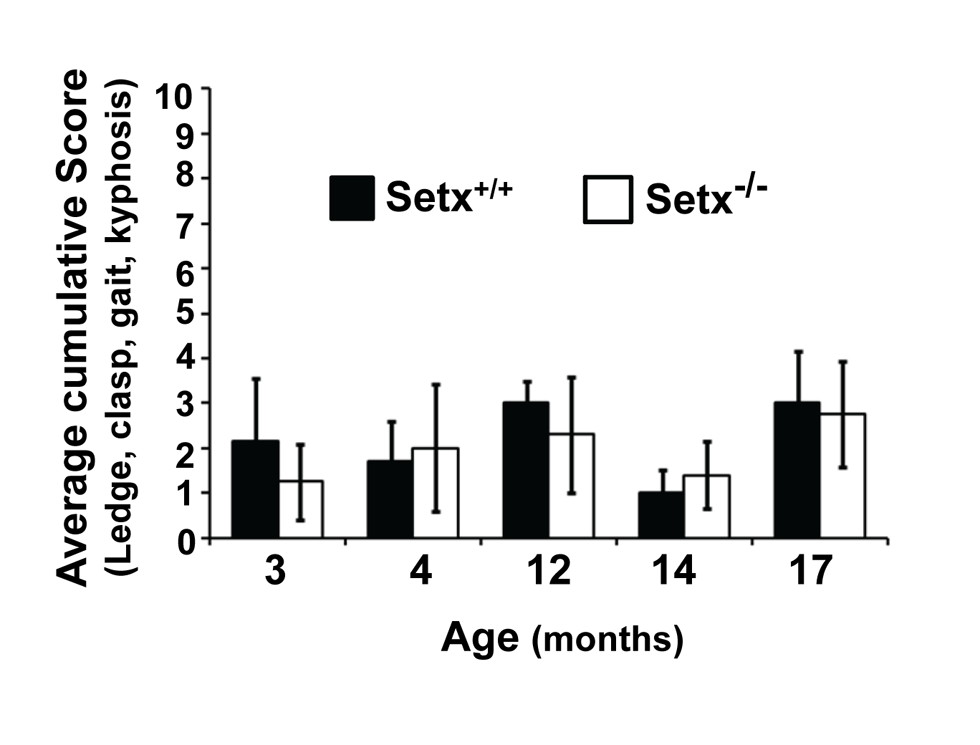

Supplement: Figure S1 — Absence of neurological phenotype and ataxia in Setx−/− mice. Neurological phenotype and ataxia were examined according to the phenotypic scoring system developed by Guyenet et al. 2010 [13]. No gross abnormal behaviour and ataxic phenotype progression was observed over a period of 3 to 17 months. Briefly, this scoring system combines phenotypic assessments that have been previously employed to assess various models of neurological disease including spinocerebellar ataxia, Huntington's disease and spinobulbar muscular atrophy [55]–[57]. Measures include hindlimb clasping, ledge test, gait and kyphosis. Each measure was recorded on a scale of 0–3 (0 representing the absence of the relevant phenotype and 3 the most severe manifestation) with a combined total of 0–12 for all four measures. Ledge test is a direct measure of coordination, the most directly comparable to human signs of cerebellar ataxia, which is impaired in cerebellar ataxias and many other neurodegenerative disorders. Hindlimb clasping is a marker of disease progression in many mouse models of neurodegeneration and certain ataxias [55]. Gait is a measure of coordination and muscle function and kyphosis is a characteristic dorsal curvature of the spine that is commonly observed in mouse models of neurodegenerative diseases [56], [57]. Mice were assessed on a 0–3 scale each for ledge test, clasping, gait and kyphosis. Average composite score for Setx+/+ and Setx−/− at each age was calculated. (TIF) [file pgen.1003435.s001.tif]

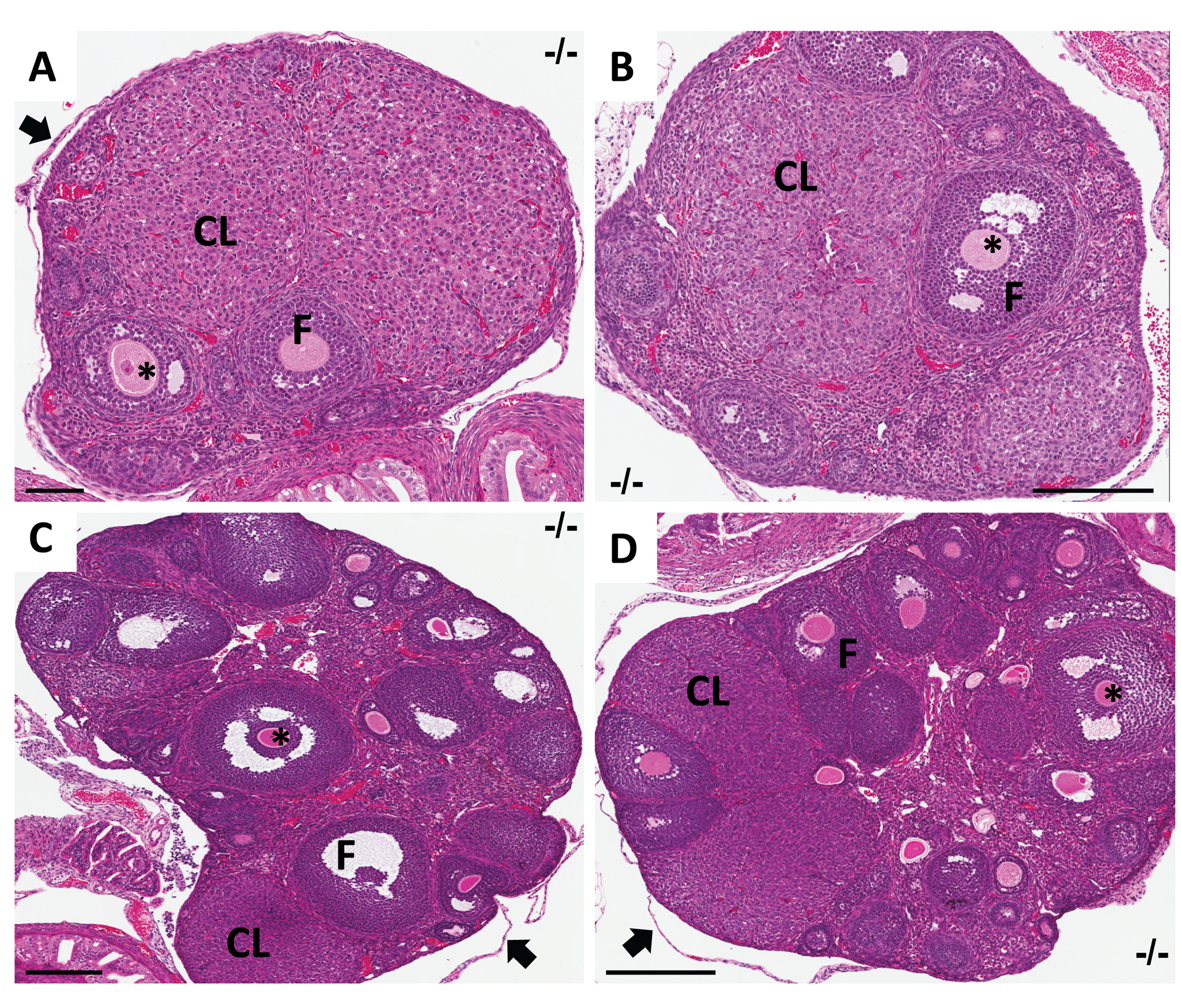

Supplement: Figure S2 — Normal ovary structure in Setx−/− mice. We examined the histology of Setx−/− ovaries at 8 months of age to determine whether they exhibited similar signs as in the human patients [1], [2]. A–D: Haematoxylin and eosin-stained 4 µm sections of four 8-month old Setx−/− mouse ovaries. Pre-antral and antral follicles (F) are present in all sections and many follicles contain oocytes (asterisks). All sections also contain corpora lutea (CL), suggesting ovulation has occurred and the hypothalamic-pituitary-gonadal endocrine axis is intact in these mice. (Arrowheads denote the position of the bursal membrane enclosing the mouse ovary.) Scale bar, 200 µm. (TIF) [file pgen.1003435.s002.tif]

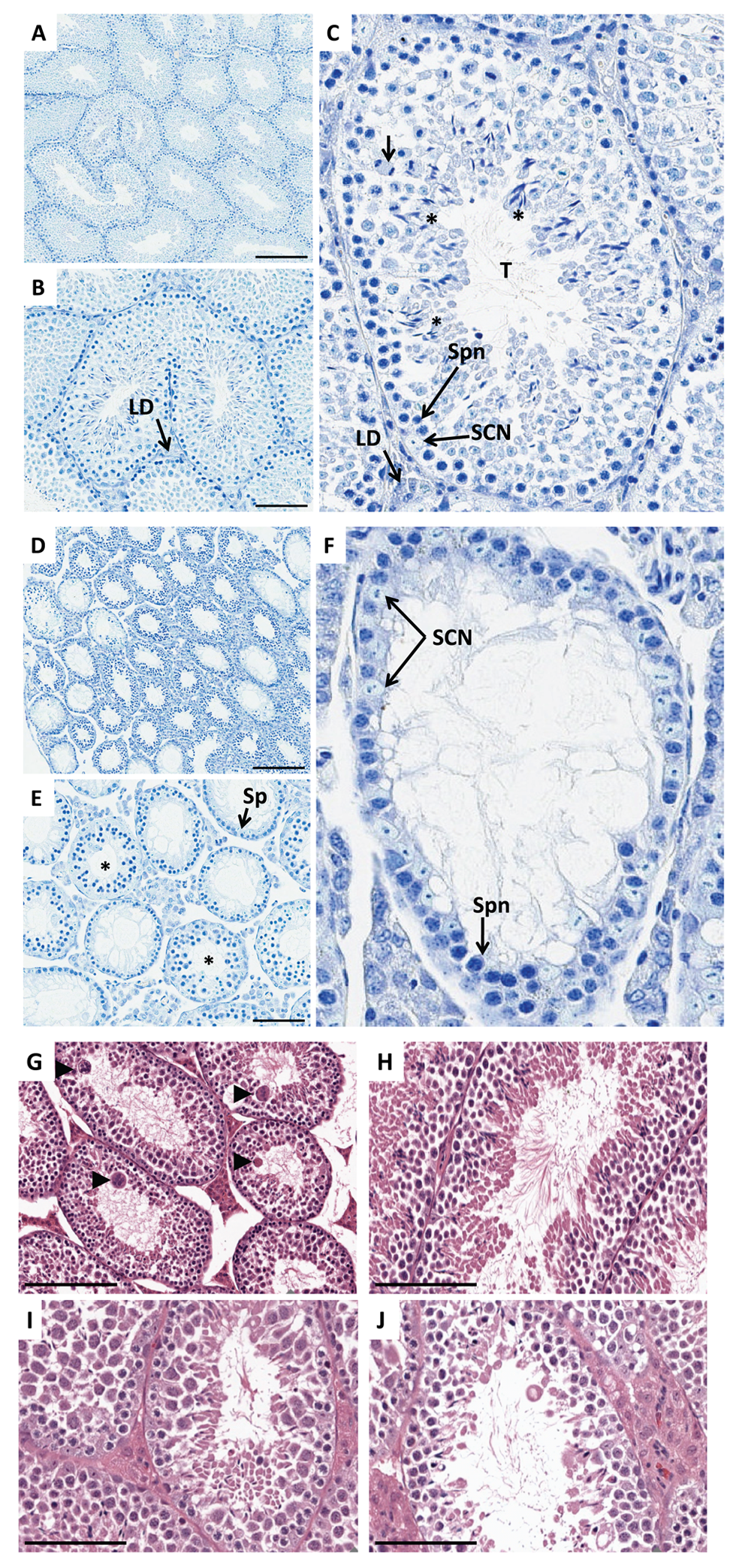

Supplement: Figure S3 — Testis histology of Setx-deficient mice. A. Testis of a 35-day old Setx+/+ mouse showing normal testis histology. Scale bar, 200 µm. B. Testis of a 35-day-old Setx+/+ mouse. Scale bar, 100 µm. Interstitial (Leydig) cells lie between the seminiferous tubules (LD). C. Seminiferous tubule from a Setx+/+ mouse at higher magnification. The tubule contains primary spermatocytes undergoing mitosis (short arrow), and elongated spermatids (asterisks) towards the lumen of the tubule, in which few sperm tails (T) can be seen. LD cells lie between the tubules. Sertoli cell nuclei (SCN) are present on the basement membrane of the tubule. D. Testis of a 35-day-old Setx−/− mouse showing seminiferous tubules with smaller diameter and disrupted spermatogenesis. Scale bar, 200 µm. E. Testis of a 35-day-old Setx−/− mouse, showing tubules at different stages of the seminiferous epithelial cycle. In some stages, spermatocytes (asterisks) are abundant. In other stages of the seminiferous cycle spermatogonia and some spermatocytes (Sp) line an almost empty tubule. Scale bar, 100 µm F. Seminiferous tubule of a 35-day-old Setx−/− mouse testis, showing spermatogonia (Spn) and Sertoli cell nuclei (SCN) lining the basement membrane of the tubule. The lumen appears to contain remnants of Sertoli cell cytoplasm. Elongated spermatids and spermatozoa are completely absent. G–J. Male Setx+/− mice exhibit signs of reduced fertility at 8 months of age. G. Seminiferous tubules contain few mature spermatozoa and large inclusions (arrowheads), suggesting increased apoptosis of spermatogenic cells. The seminiferous epithelium contains post-meiotic round and elongating spermatids. Scale bar, 200 µm. H. Seminiferous tubule from an 8-month Setx+/− mouse testis; Scale bar, 100 µm. The seminiferous epithelium contains all stages of spermatogenesis, including round (R) and elongating spermatids (asterisks) and there are sperm tails (T) in the lumen. I. Seminiferous tubule from an 8-month Setx+/− mouse testis. [file pgen.1003435.s003.tif]

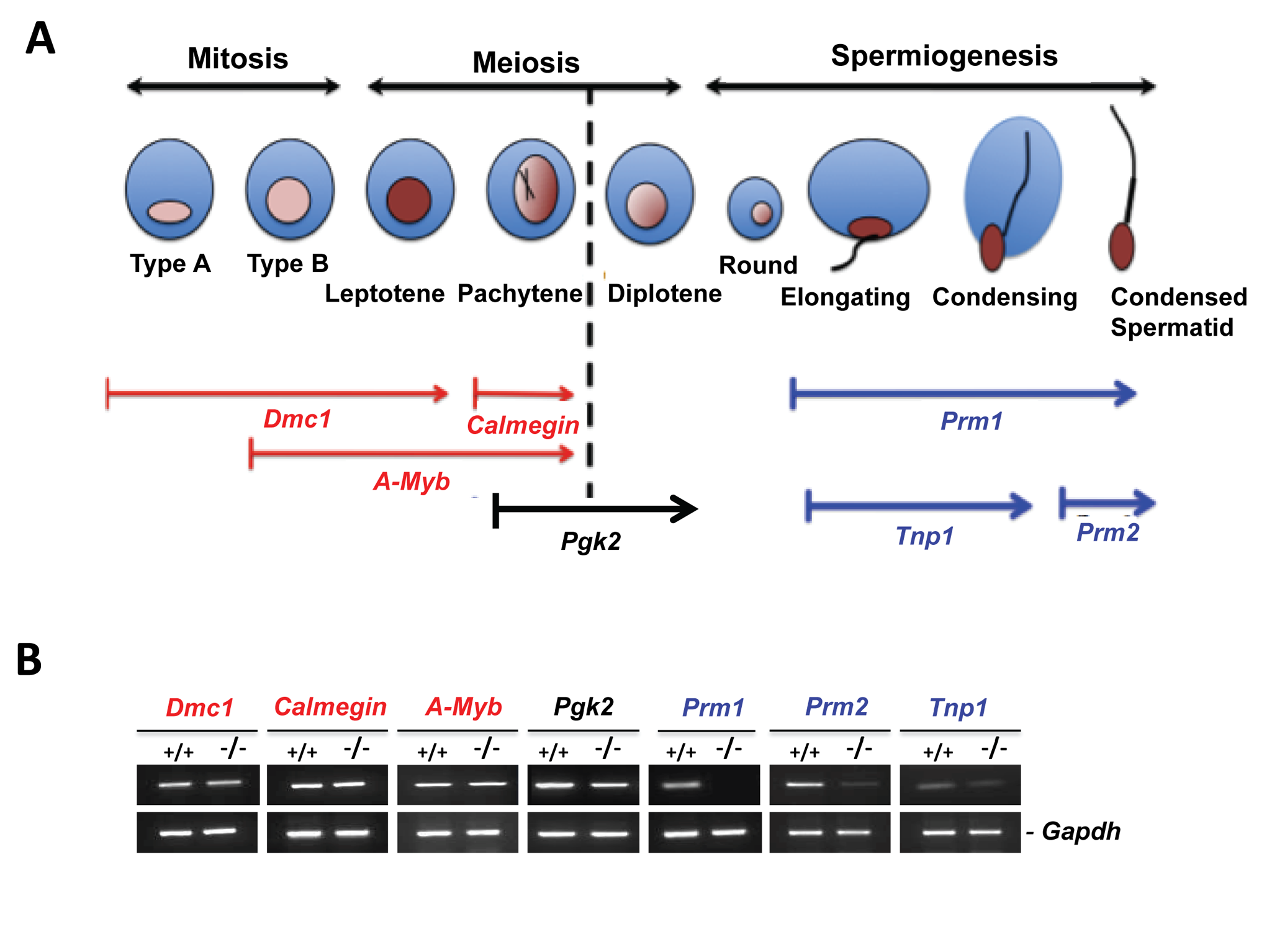

Supplement: Figure S4 — Abortion of meiosis following a block at pachytene stage in Setx−/− mice. A. Schematic representation of the various stages of spermatogenesis and the temporal expression of stage-specific makers as previously reported [58]. B. Semi-quantitative RT-PCR analysis of spermatogenesis stage-specific markers in Setx+/+ and Setx−/− testes. Similar levels of expression for mitosis and meiosis-specific markers (Dmc1, Calmegin, A-myb) were observed in both Setx+/+ and Setx−/− testes. A reduction of Pgk2 is noticable in Setx−/−. A marked reduction in expression for post-meiotic germ cells (Prm1, Prm2 and Tnp1) is observed in Setx−/− testes in agreement the absence of these cells in Setx−/− seminiferous tubules as shown in Figure 2. These data suggest that Setx−/− spermatocytes do not proceed past meiosis. Gapdh was used as an internal standard. Cal, Calmegin. (TIF) [file pgen.1003435.s004.tif]

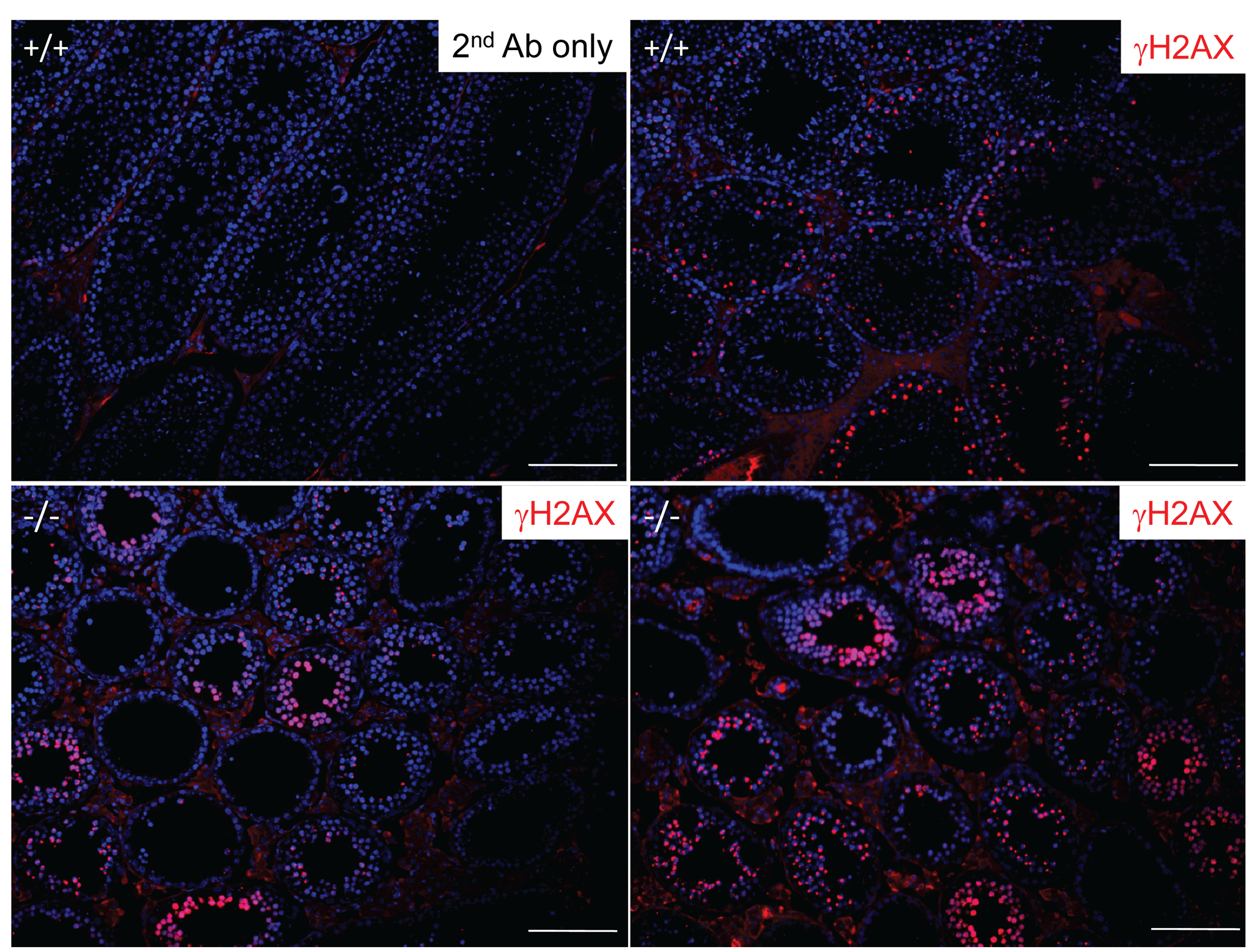

Supplement: Figure S5 — Elevated levels of DSBs in adult Setx−/− testes sections. γH2AX staining of adult testes histological sections highlight the extensive amount of DNA DSB breaks in Setx−/− seminiferous tubules. (TIF) [file pgen.1003435.s005.tif]

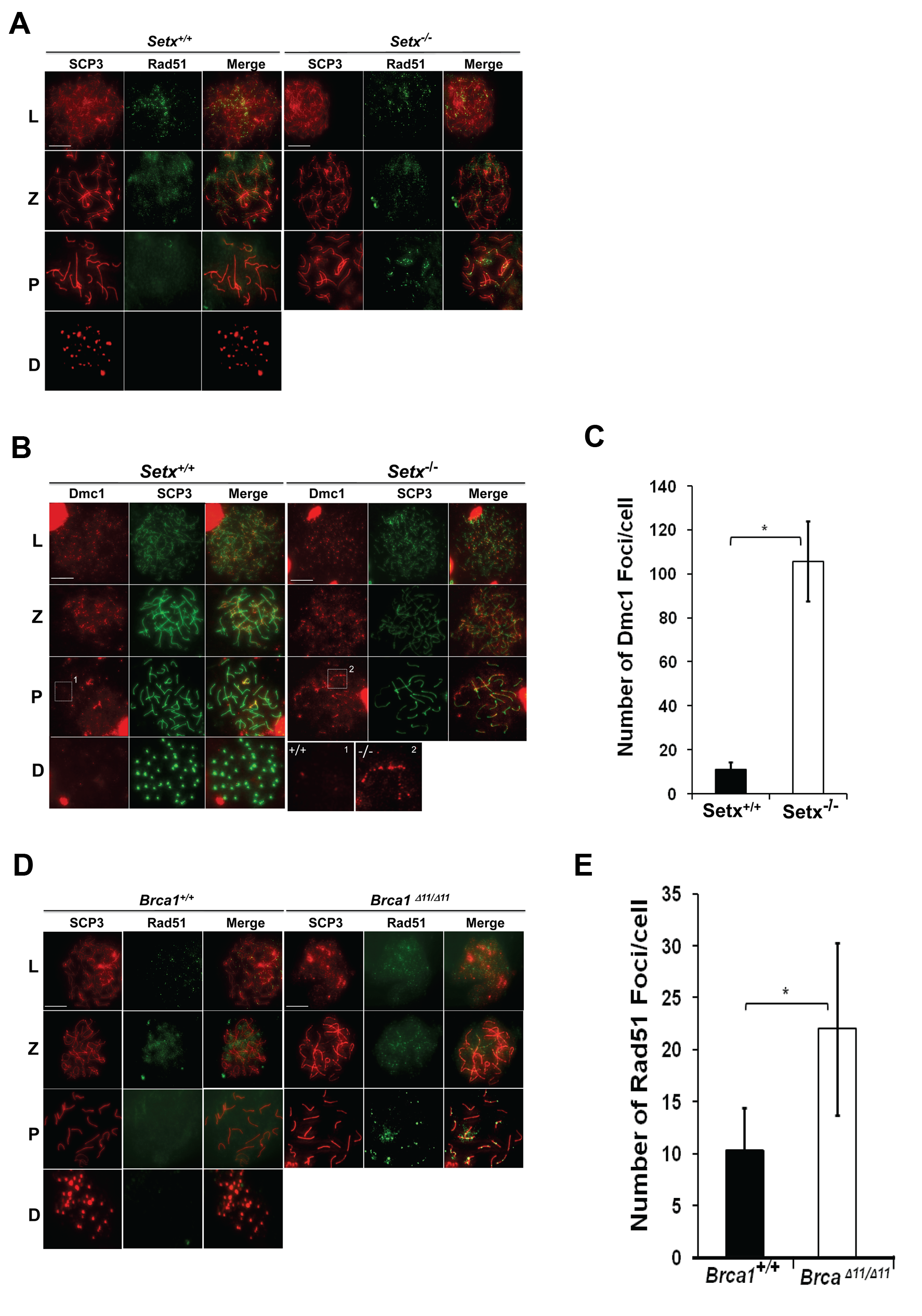

Supplement: Figure S6 — Persistance of Rad51 and Dmc1 foci at pachytene stage in Setx−/− and Brca1Δ11/Δ11p53+/− spermatocytes. A. Persistence of DSB repair intermediates in pachytene cells of Setx−/− mice. Normal Rad51 foci formation occurred at leptotene and zygotene stage in Setx+/+ and Setx−/− mice, there was persistence of Rad51 foci at pachytene stage in Setx−/− spermatocytes indicating the presence of unrepaired DSBs. Scale bar, 20 µm. B. Persistence of DSB repair intermediates in pachytene cells of Setx−/− mice. Normal Dmc1 foci formation occurred at leptotene and zygotene stage in Setx+/+ and Setx−/− mice, there was persistence of Dmc1 foci at pachytene stage in Setx−/− spermatocytes indicating the presence of unrepaired DSBs (compare 1 and 2). Scale bar, 20 µm. C. Quantitation of Dmc1 foci at pachytene stage in Setx+/+ and Setx−/− mice. A 10-fold increase of Dmc1 foci was observed in Setx−/−. (Student's t-test, n = 50) * indicates p<0.05. D. Rad51 foci in Brca1+/+p53+/− and Brca1Δ11/Δ11p53+/−. Normal Rad51 foci formation occurred at leptotene and zygotene stage in Setx+/+ and Setx−/− mice, there was persistence of Rad51 foci at pachytene stage in Brca1Δ11/Δ11p53+/− spermatocytes indicating the presence of unrepaired DSBs. Scale bar, 20 µm. E. Quantitation Rad51 foci at pachytene stage in Brca1+/+p53+/− and Brca1Δ11/Δ11p53+/− spermatocytes. A 2.5-fold increase in the numbers of Rad51 foci was observed in Brca1Δ11/Δ11p53+/− (Student's t-test, n = 50). * indicates p<0.05. (TIF) [file pgen.1003435.s006.tif]

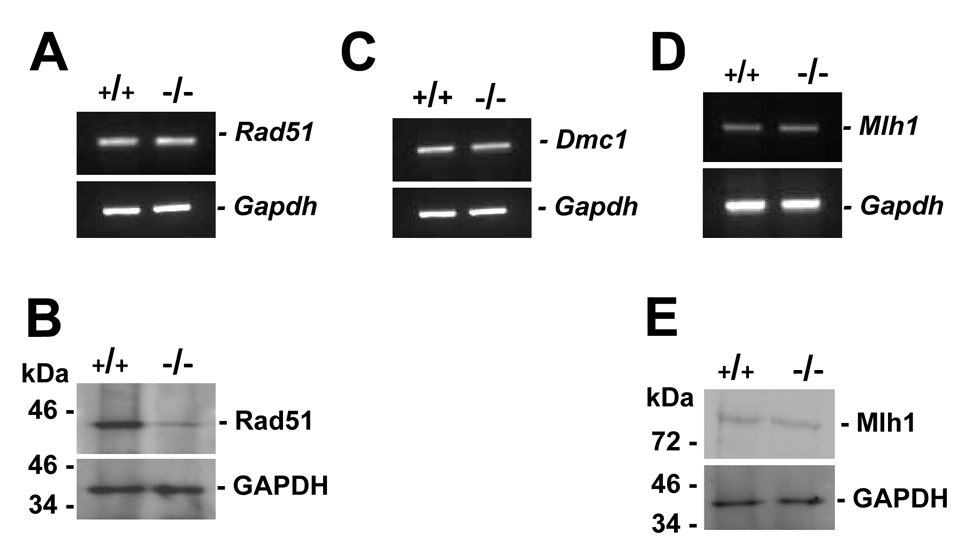

Supplement: Figure S7 — Expression levels of recombination factors. A. RT-PCR analysis of Rad51 expression revealed that the increased number of Rad51 foci in Setx−/− is not due to an increased expression of Rad51 gene since similar levels of Rad511 mRNA levels were detected in Setx+/+ and Setx−/− testes. B. Immunoblotting of Setx+/+ and Setx−/− testes protein extracts with anti-Rad51 antibody shows reduced levels of Rad51 protein in Setx−/−. Anti-GAPDH was used as a loading control. C. RT-PCR analysis of Dmc1 expression revealed that the increased number of Dmc1 foci in Setx−/− is not due to an increased expression of Dmc1 gene since similar levels of Dmc1 mRNA levels were detected in Setx+/+ and Setx−/− testes. D. RT-PCR analysis of Mlh1 expression revealed that the absence of Mlh1 foci in Setx−/− is not due to a lack of expression of Mlh1 gene since similar levels of Mlh1 mRNA levels were detected in Setx+/+ and Setx−/− testes. E. Immunoblotting of Setx+/+ and Setx−/− testes protein extracts with anti-Mlh1 antibody shows similar levels of Mlh1 protein. Anti-GAPDH was used as a loading control. (TIF) [file pgen.1003435.s007.tif]

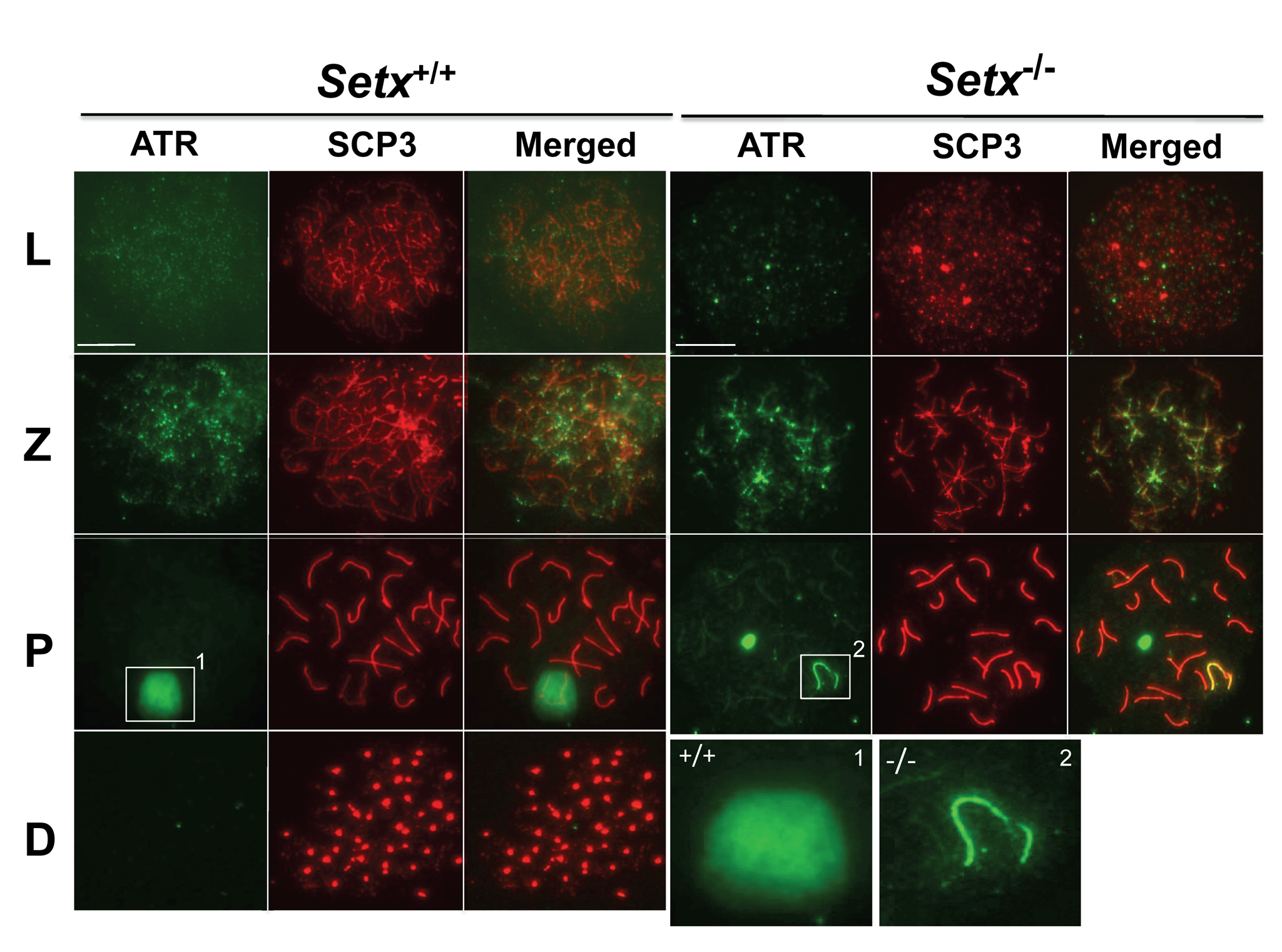

Supplement: Figure S8 — Abnormal localisation of ATR in Setx−/− pachytene spermatocytes. ATR foci formation occurred at leptotene and zygotene stages in Setx+/+ and Setx−/− spermatocytes, however, ATR failed to spread to XY chromatin domain in Setx−/− at pachytene stage. Scale bar, 20 µm. (TIF) [file pgen.1003435.s008.tif]
