# Supplementary material for: Senataxin Plays an Essential Role with DNA Damage Response Proteins in Meiotic Recombination and Gene Silencing
Source: PLoS Genet. 2013 Apr 11;9(4):e1003435. doi: 10.1371/journal.pgen.1003435 (PMC3623790; doi:10.1371/journal.pgen.1003435)
Supplement: Table S1 — Sequences of primers used for the gene expression analysis. Sequences of the primers used for the spermatogenesis stage specific markers and X- and Y-linked gene expression analysis. (PDF) [file pgen.1003435.s009.pdf]

Table S1. Primers used in gene expression analysis.

Mlh1F	5'-AGGAGCTGATGCTGAGGC-3'
Mlh1R	5'-AGGAGCTGATGCTGAGGC-3'
Dmc1F	5'-TTCGTACTGGAAAAACTCAGCTGTATC-3'
Dmc1R	5'-TTCGTACTGGAAAAACTCAGCTGTATC-3'
CalmeginF	5'-ATATGCGTTTCCAGGGTGTTGGAC-3'
CalmeginR	5'-ATATGCGTTTCCAGGGTGTTGGAC-3
Pgk2F	5'-CTGTTGCTGATGAGCTCAAG-3'
Pgk2R	5'-CTGTTGCTGATGAGCTCAAG-3'
A-mybF	5'-AAGAAGTTGGTTGAACAACACGG-3'
A-mybR	5'-AAGAAGTTGGTTGAACAACACGG-3'
Scp1F	5'-ATGGAGAAGCAAAAGCCCTTC-3'
Scp1R	5'-ATGGAGAAGCAAAAGCCCTTC-3'
Scp3F	5'-GGTGGAAGAAAGCATTCTGG-3'
Scp3R	5'-GGTGGAAGAAAGCATTCTGG-3'
Prm1F	5'-ATGCTGCCGCAGCAAAAGCA-3'
Prm1R	5'-ATGCTGCCGCAGCAAAAGCA-3'
Prm2F	5'-ATGGTTCGCTACCGAATGAGG-3'
Prm2R	5'-ATGGTTCGCTACCGAATGAGG-3'
Rad51F	5'-AAGTTTTGGTCCACAGCCTATTT-3'
Rad51R	5'-AAGTTTTGGTCCACAGCCTATTT-3'
Tnp1F	5'-ACCAGCCGCAAGCTAAAGAC-3'
Tnp1FR	5'-ACCAGCCGCAAGCTAAAGAC-3'
GapdhF	5'-ATTGTCAGCAATGCATCCTG-3'
GapdhR	5'-ATTGTCAGCAATGCATCCTG-3'
ActbF	5'- GCGGACTGTTACTGAGCTGCGT-3'
ActbR	5'- GAAGCAATGCTGTCACCTTCCC-3'
Ubey1F	5'- ATTGACTTTGAGAAGGATGAC-3'

Ubey1R	5’- CAGACACACAAGGCCAACTAT-3’
Ube1xF	5’- GTGCATTCCCCTAAGCCCCA-3’
Ube1xR	5’- GGGTAATTATCCTTTTATTGGGAT-3’
RbmyF	5’- AACCGAAGTAACATATACTCA-3’
RbmyR	5’- ATCTGCTTTCTCCACGACCTC-3’
Fthl17F	5’- TACTTTGACCGTGATGACGTG-3’
Fthl17R	5’- AGTTTTGCTCCAGGAAATGGC-3’
Usp26F	5’- AATGTAACGAAGGGAGAAGTG-3’
Usp26R	5’- AGGCTTTGCCTTCTTATCGAG-3’
Tktl1F	5’- TCAAAGGGACTACCATTTGTT-3’
Tktl1R	5’- AACAGGGGGCGAAGTCATACA-3’
DazlF	5’- TTCAGGCATATCCTCCTTATC-3’
DazlR	5’- ATGCTTCGGTCCACAGACTTC-3’
SetxF	5’- CCGGAATTCATTATTAAGACCAGTGACCC-3’
SetxR	5’- GATGGCCTCGAGCTATAGAAATTGTCTCCTC-3’
